# Innate Immune Responses and Rapid Control of Inflammation in African Green Monkeys Treated or Not with Interferon-Alpha during Primary SIVagm Infection

**DOI:** 10.1371/journal.ppat.1004241

**Published:** 2014-07-03

**Authors:** Béatrice Jacquelin, Gaël Petitjean, Désirée Kunkel, Anne-Sophie Liovat, Simon P. Jochems, Kenneth A. Rogers, Mickaël J. Ploquin, Yoann Madec, Françoise Barré-Sinoussi, Nathalie Dereuddre-Bosquet, Pierre Lebon, Roger Le Grand, François Villinger, Michaela Müller-Trutwin

**Affiliations:** 1 Institut Pasteur, Regulation of Retroviral Infection Unit, Paris, France; 2 Paris Diderot University, Sorbonne Paris Cité, Paris, France; 3 Division of Pathology, Yerkes National Primate Research Center, Emory University, Atlanta, Georgia, United States of America; 4 Institut Pasteur, Emerging Diseases Epidemiology Unit, Paris, France; 5 CEA, Division of Immuno-Virology, DSV, iMETI, Fontenay-aux-Roses, France; 6 Saint-Vincent de Paul Hospital & Paris Descartes University, Paris, France; Vaccine Research Center, United States of America

## Abstract

Chronic immune activation (IA) is considered as the driving force of CD4^+^ T cell depletion and AIDS. Fundamental clues in the mechanisms that regulate IA could lie in natural hosts of SIV, such as African green monkeys (AGMs). Here we investigated the role of innate immune cells and IFN-α in the control of IA in AGMs. AGMs displayed significant NK cell activation upon SIVagm infection, which was correlated with the levels of IFN-α. Moreover, we detected cytotoxic NK cells in lymph nodes during the early acute phase of SIVagm infection. Both plasmacytoid and myeloid dendritic cell (pDC and mDC) homing receptors were increased, but the maturation of mDCs, in particular of CD16^+^ mDCs, was more important than that of pDCs. Monitoring of 15 cytokines showed that those, which are known to be increased early in HIV-1/SIVmac pathogenic infections, such as IL-15, IFN-α, MCP-1 and CXCL10/IP-10, were significantly increased in AGMs as well. In contrast, cytokines generally induced in the later stage of acute pathogenic infection, such as IL-6, IL-18 and TNF-α, were less or not increased, suggesting an early control of IA. We then treated AGMs daily with high doses of IFN-α from day 9 to 24 post-infection. No impact was observed on the activation or maturation profiles of mDCs, pDCs and NK cells. There was also no major difference in T cell activation or interferon-stimulated gene (ISG) expression profiles and no sign of disease progression. Thus, even after administration of high levels of IFN-α during acute infection, AGMs were still able to control IA, showing that IA control is independent of IFN-α levels. This suggests that the sustained ISG expression and IA in HIV/SIVmac infections involves non-IFN-α products.

## Author Summary

Chronic inflammation is considered as directly involved in AIDS pathogenesis. The role of IFN-α as a driving force of chronic inflammation is under debate. Natural hosts of SIV, such as African green monkeys (AGMs), avoid chronic inflammation. We show for the first time that NK cells are strongly activated during acute SIVagm infection. This further demonstrates that AGMs mount a strong early innate immune response. Myeloid and plasmacytoid dendritic cells (mDCs and pDCs) homed to lymph nodes; however mDCs showed a stronger maturation profile than pDCs. Monitoring of cytokine profiles in plasma suggests that the control of inflammation in AGMs is starting earlier than previously considered, weeks before the end of the acute infection. We tested whether the capacity to control inflammation depends on the levels of IFN-α produced. When treated with high doses of IFN-α during acute SIVagm infection, AGMs did not show increase of immune activation or signs of disease progression. Our study provides evidence that the control of inflammation in SIVagm infection is not the consequence of weaker IFN-α levels. These data indicate that the sustained interferon-stimulated gene induction and chronic inflammation in HIV/SIVmac infections is driven by factors other than IFN-α.

## Introduction

Chronic immune activation during HIV infection is considered as the main driver of CD4^+^ T cell depletion and AIDS, and early T cell activation is a better predictor of the outcome of the infection than viral load [1]. Recent observations suggest that inflammation is even more important than T cell activation to predict disease progression and mortality [2], [3]. Already in the acute primary phase of HIV-1 infection, the levels of soluble inflammatory mediators, such as IP-10 (CXCL10), were predictive of disease progression [4], [5].

Type I IFN (IFN-I), such as IFN-α, is an important component of innate immunity providing a first-line defense to viral infections, as well as bridging the innate and adaptive immune systems. This cytokine is mainly produced by plasmacytoid dendritic cells (pDCs) in viral infections. These cells interact with myeloid dendritic cells (mDCs), NK cells, monocytes, T and B cells and contribute to the orchestration of the immune response. IFN-α production is critical for the activation of NK cells, enhancing IFN-γ secretion and their cytotoxicity. Reciprocally, NK cells can affect pDC maturation and function [6]. Thus, upon infection, a crosstalk is engaged between NK cells, pDCs and mDCs, an interplay that involves IFN-I activity coupled with the release of other soluble factors [7].

Upon recognizing HIV-1, pDCs become activated, secreting high amounts of IFN-α and inflammatory cytokines, such as TNF-α [8]. This leads to bystander maturation of mDCs [9]. Both pDCs and mDCs are reduced in number and function in HIV-1 infected individuals in the circulation [10]. PDCs have been shown to migrate to lymph nodes (LNs), gut and spleen and accumulate there [11]–[14]. As a matter of fact, the diminished responses seen in disease progressors might be explained by pDC exhaustion or trafficking to tissues [13], [15]. Moreover, a defect in the pDC-NK cell cross-talk, due in large part to impaired NK cell responsiveness to IFN-α, has been described in HIV-1 infection [16], [17]. Still the role of IFN-α in HIV infection is controversial. On the one hand, IFN-α may delay disease progression by inhibiting viral replication through the induction of cellular restriction factors and by stimulating various components of the immune response involved in the control of HIV [18], [19]. A beneficial effect of IFN-α is also suggested by the observation of higher levels of pDCs and IFN-α production by TLR9-stimulated pDCs in HIV-infected long-term non-progressors [20]. On the other hand, IFN-α levels and type I interferon-stimulated gene (ISG) are markedly increased and sustained in progressors as compared to long-term non-progressors [21], [22]. Indeed, in HIV-untreated patients, high levels of ISG, such as IP-10, were associated with a more rapid CD4^+^ T cell depletion [4], [23]. Thus, it has been suggested that IFN-α might exert deleterious effects through various mechanisms. It could fuel chronic immune activation by the induction of ISGs including chemokines able of attracting target cells to the site of viral replication [24]. It could also stimulate innate immune cells, such as NK cells, which will in turn produce cytokines (IFN-γ,…) and chemokines, and indirectly contribute to the activation of other cell types. Moreover, the up-regulation of the ISG TRAIL may induce apoptosis of uninfected CD4^+^ T lymphocytes [25]. Chronic high levels of IFN-α could also induce defects in the thymopoiesis and bias in T cell selection, thereby accelerating disease progression [26].

Fundamental clues regarding the role of inflammation in AIDS and the mechanisms that protect against it may lie in natural hosts of SIV, such as African green monkeys (AGMs) and sooty mangabeys (SMs), which are asymptomatic carriers of SIV [27], [28]. This protection against AIDS is seen despite virus replication levels in blood and gut similar to HIV-1 infected humans and SIVmac-infected macaques [29]. It is associated with an absence of chronic immune activation, lacking both chronic T cell activation and chronic inflammation [27], [30]–[32]. This is not due to ignorance of the virus or to a functional defect of pDCs in sensing the virus [33]–[36]. Indeed, a vigorous innate immune response is triggered upon infection [34], [36]–[40]. Thus, the acute phase of SIVagm infection is characterized by the recruitment of pDCs to LNs, IFN-α production, induction of ISG and corresponding protein (ISP) expression [34], [36]–[40]. The levels of ISP strongly correlated with IFN-α levels during the acute phase of SIVagm infection [36]. However, there are major differences as compared to SIVmac infection: the levels of IFN-α produced in blood and LN were lower than those observed in SIVmac infection [35], [36], [38]. Moreover, in some reports, most cytokines were produced only to moderate levels in natural hosts and several pro-inflammatory cytokines were not induced at all, in contrast to the cytokine storm seen during pathogenic HIV-1/SIVmac infections [30], [35], [36], [38], [41]–[43]. Finally, ISGs, cytokines and T cell activation are down-regulated by the end of the acute phase in natural hosts and maintained as such. Thus, while in HIV/SIVmac pathogenic infections, immune activation persists, in natural hosts there are mechanisms that either prevent the onset of sustained inflammation or mechanisms that rapidly and efficiently turn them off.

In this report, we investigated the effect of SIVagm infection in AGM on innate immune cell compartments, in particular pDCs, mDCs and NK cells, and tested whether exogenous administration of IFN-α would modify the development of antiviral responses, promote chronic inflammation and/or alter clinical parameters.

## Results

### Close follow up of viral replication and T cell dynamics

**Figure 1:**
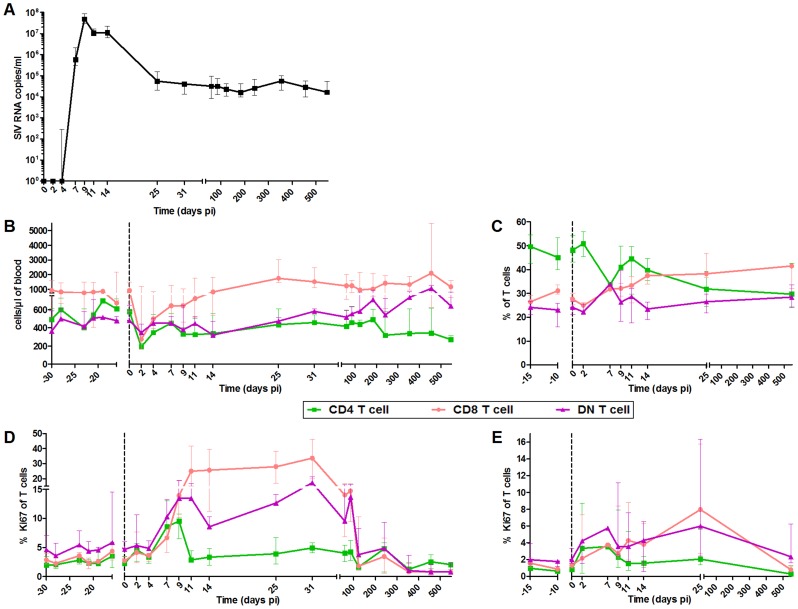
Follow-up of virological and immunological markers during primary SIVagm infection. (A) Plasma viral RNA copy numbers were measured by real-time PCR in 6 AGMs infected with SIVagm.sab92018. Flow cytometry evaluation of (B) CD4^+^, CD8^+^ and DN T cell counts in blood, (C) CD4^+^, CD8^+^ and DN T cell percentages in LNs; CD4^+^, CD8^+^ and DN T cell proliferation as evaluated by Ki-67^+^ cells percentages in (D) blood and (E) LNs. Data are presented as medians and interquartile ranges. Day zero represents the median of all the time points before infection. When p-values between time points were determined (cf. results), the Wilcoxon matched-pairs signed rank test was used.

The innate immune responses were followed in six SIVagm.sab92018-infected AGMs between days 2 and 547 post-infection (pi). We analyzed both blood and LNs. It is crucial indeed to study LNs because these are the sites where T and B cell responses are induced, shaped, and regulated and where correlates of protection were identified [44], [45]. Consistent with previous reports, AGMs displayed high levels of SIV replication with a peak on day 9 pi coinciding with a transient decline in CD4^+^ T cell levels (Figure 1A and B) [30], [36], [46]. We monitored T cell proliferation and confirmed that the primary phase of SIVagm infection in AGMs is associated with a transient increase in the percentages of Ki-67^+^ T cells in blood and LNs (Figure 1D and E) [30]. The peak of Ki-67^+^ CD4^+^ T cells was observed between day 7 and 9 pi (at day 9, p = 0.031), while the percentage of Ki-67^+^ CD8^+^ T cells reached a plateau on day 11 pi in blood (p = 0.008) and a peak on day 25 pi in LN (p = 0.016). The Ki-67^+^ CD4^+^ and DN T cell frequencies subsequently decreased on day 11 and that of Ki-67^+^ CD8^+^ T cells after day 31 pi.

### PDCs from SIVagm-infected AGMs displayed a more immature phenotype as compared to mDCs

**Figure 2:**
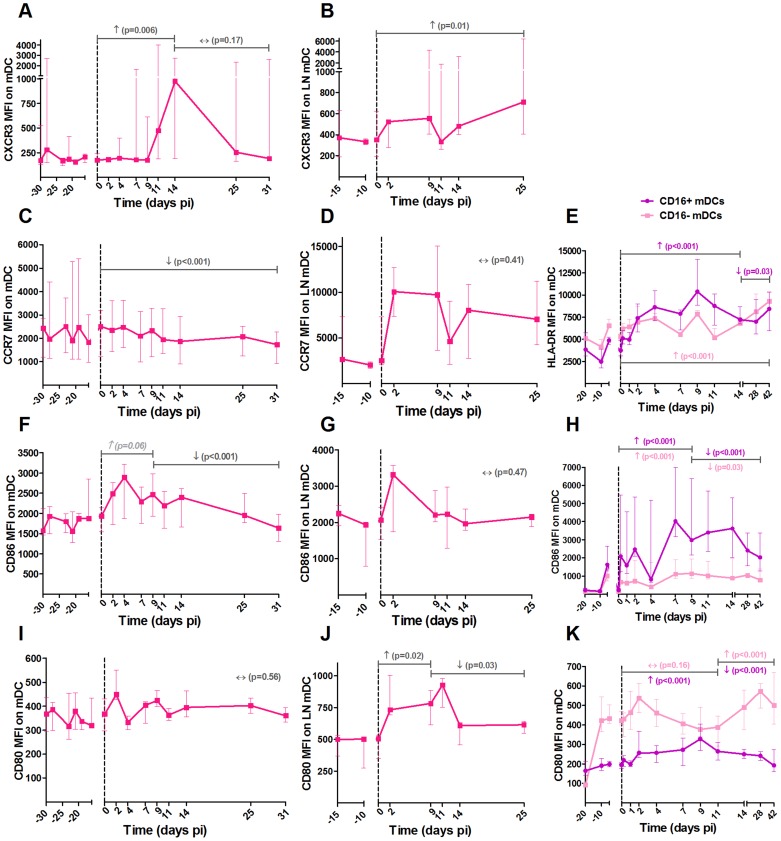
Expression of homing and maturation marker on mDCs in blood and lymph nodes following SIVagm infection. MDCs were defined as shown in Figure S1. Expression levels of the chemokine receptors (A, B) CXCR3 and (C, D) CCR7 and the co-stimulatory (F, G) CD86 and (I, J) CD80 molecules on mDCs by flow cytometry in blood (left panels) and LNs (center panels) are shown as mean fluorescence intensity (MFI) after isotype-control levels were subtracted (n = 6 AGMs). A phenotypic analysis of mDC subsets was performed on another group of 8 AGMs (right panels). CD16 was added to differentiate the subsets. The expression levels of (E) HLA-DR, (H) CD86 and (K) CD80 on CD16^+^ (in purple) and CD16^−^ (in pink) mDCs are shown as MFI. Data are presented as medians and interquartile ranges. Day zero represents the median of all the time points before infection. P-values were obtained from a linear mixed effect model to characterize each marker's progression (with a logarithm transformation for panel A and B).

**Figure 3:**
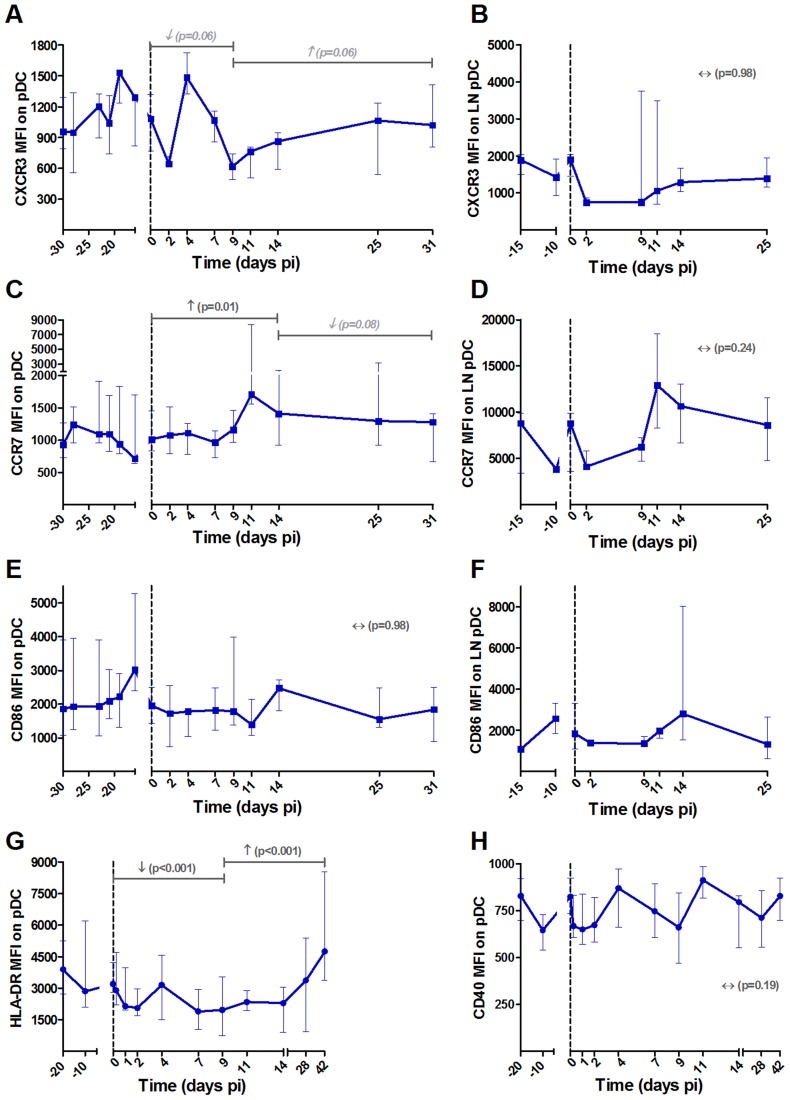
Follow-up of homing and maturation markers on pDCs in blood and lymph nodes following SIVagm infection. PDCs were defined as shown in Figure S1. Expression levels of the chemokine receptors (A, B) CXCR3 and (C, D) CCR7 and the (E, F) co-stimulatory CD86 on (A, C, E) blood and (B, D, F) LN pDCs are shown as MFI after substraction of isotype-control values (n = 6 AGMs). The expression levels of (G) HLA-DR and (H) CD40 were analyzed on another group of 8 AGMs. PDCs were here defined as CD20^−^ BDCA2^+^ CD123^+^ HLA-DR^+^. Data are presented as medians and interquartile ranges. Day zero represents the median of all the time points before infection. See legend to Figure 2 for a description of p-values (logarithm transformation for panel B).

**Figure 4:**
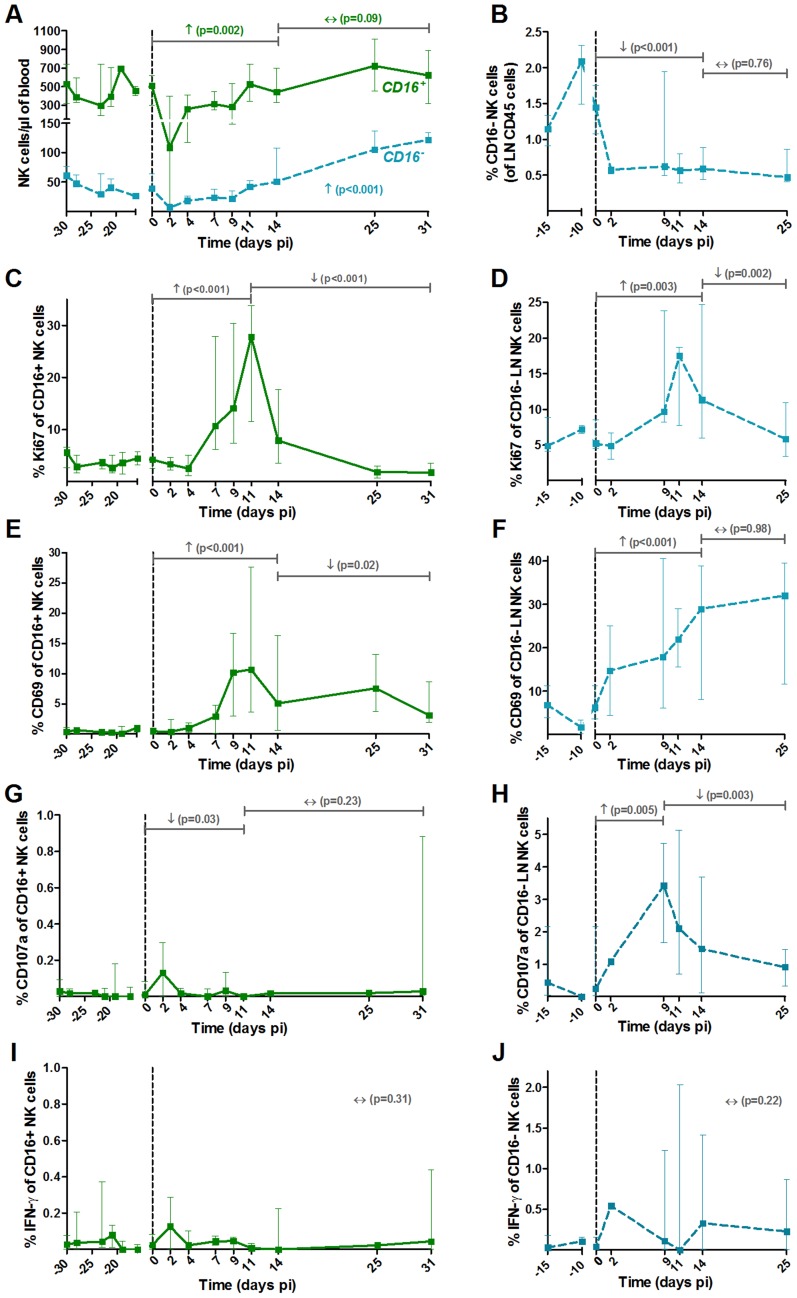
NK cell activation pattern and function in blood and lymph nodes upon SIVagm infection. NK cells were defined as CD45^+^ Lineage^−^ (CD3, CD20, HLA-DR) CD8α^+^ NKG2A^+^ CD16^+/−^. The major NK cell populations were CD16^+^ in blood and CD16^−^ in LNs (Figure S1). (A) Absolute numbers of NK cells in blood and (B) frequencies among CD45^+^ LN cells throughout SIVagm infection. Frequencies of (C, D) proliferating (Ki-67^+^) and (E, F) activated (CD69^+^) NK cells in blood (left panels) and LNs (right panels). Cytotoxicity was estimated by measuring the frequency of degranulating cells (CD107a^+^ cells) in (G) blood and (H) LNs. The level of cytokine production was assessed by direct *ex vivo* intra-cellular staining of the frequency of IFN-gamma in NK cells from (I) blood and (J) LN. Data are presented as medians and interquartile ranges (n = 6 AGMs). Day zero represents the median of all the time points before infection. See legend to Figure 2 for a description of p-values (logarithm transformation for panel B, C and G).

To better understand the trafficking and function of mDCs, pDCs and NK cells in AGMs, we investigated the early changes in activation, maturation, function and homing markers of these cells in blood and LNs (Figures 2, 3 and 4, respectively). The gating strategy used for flow cytometry analysis is depicted in Figure S1. We first confirmed previous data on pDC and mDC dynamics during SIVagm infection (data not shown) [38], [47], [48]. We then studied two homing receptors for DCs: the homing inflammatory chemokine receptor CXCR3, which is the receptor for CXCL9, IP-10 and CXCL11 and CCR7, which is a receptor for chemokines that are expressed constitutively in secondary lymphoid organs. In line with the increase of mDC frequency in LNs, expression of CXCR3 increased on these cells in blood and LNs during acute infection (Figure 2A and B). Moreover, the percentage of CCR7^+^ mDCs was transiently increased in blood (p = 0.039 at day 9 pi) (not shown) while CCR7 levels on mDC surface did not increase (Figure 2C and D). For pDCs, the expression levels of CCR7 were significantly increased up to day 14 pi, while the CXCR3 levels were not increased (Figure 3A–D). Hence, CXCR3 and CCR7 showed opposite expression profiles on pDCs and mDCs. Still, both mDCs and pDCs showed an increase expression of one homing marker, concomitant with their increases in LNs [38], [47], [48]

MDCs showed up-regulations of the maturation markers CD80 and CD86 in blood and LN at early time points of primary infection (in blood: p = 0.008 at day 4 pi for CD86 and p = 0.008 at day 2 pi for CD80, in LNs: p = 0.031 at day 9 for CD80) (Figure 2F, G, I and J). In contrast, the expression of CD86 was not modulated on pDCs (Figure 3E and F).

It was surprising to see this discrepancy in maturation profiles between mDCs and pDCs. To confirm these findings, we analyzed the maturation profiles of mDCs and pDCs in the blood of another group of 8 AGMs infected with SIVagm. In this group, we further distinguished CD16^+^ mDCs (inflammatory) and CD16^−^ mDCs (Figure 2E, H and K). In addition to the maturation markers CD80 and CD86, we also measured HLA-DR expression. The two CD16^+^ and CD16^−^ mDC subsets were present in similar frequencies in blood (not shown). Both mDC subsets displayed increases in the expression of CD80, CD86 and HLA-DR. These maturation markers were more significantly increased on the CD16^+^ than on the CD16^−^ subset (Figure 2E, H and K).

We confirmed the pDC phenotype in these 8 additional animals by staining for BDCA-2 (Figure 3G–H). We choose to follow HLA-DR and CD40 as these markers are well known to be up-regulated when pDCs mature and CD40 expression is increased on pDCs in SIV/HIV pathogenic infection [49], [50]. On AGM pDCs, the expression of HLA-DR was significantly down-regulated during the acute phase and the expression of the activation/maturation marker CD40 was not modulated (Figure 3G and H). The expression of CCR7 was transiently increased (p = 0.031 at day 4 pi) in this group of AGM too (not shown). These analyses thus confirm that mDCs show a more pronounced maturation profile than pDCs during SIVagm infection.

### Strong activation of NK cells during primary SIVagm infection

The two main functional NK cell subsets (cytolytic versus cytokine producers) were analyzed. These two subsets were differentiated based on the expression of CD16, the CD16^+^ subset being the predominant one in blood, as in humans and macaques (Figure 4A). Similar to cynomolgus macaques, the CD56 marker cannot be used in AGM to differentiate the NK cell subsets [51]. Thus, NK cells were defined as CD3^−^ CD20^−^ HLA-DR^−^ CD8α^+^ NKG2A^+^ CD16^+/−^ (Figure S1B and C), as in other studies on NK cells from macaques and SMs [39], [52]. A significant transient decline of both subsets was observed in blood (p = 0.031 at day 2 pi) (Figure 4A). NK cell numbers then progressively increased to reach 249% of the pre-infection levels at the end of primary infection (day 25–31 pi) for the major CD16^+^ subset, and 154% for the CD16^−^ subset. They returned to baseline levels in the chronic phase (not shown). In LNs, only few NK cells were detectable and most corresponded to the CD16^−^ subset, similar to humans [53]. A significant decrease in the percentage of the CD16^−^ subset in LNs was observed (Figure 4B). The levels of CD16^+^ cells in LNs were too low to be followed. Thus, CD16^+^ NK cells in the blood exhibited maximal increase at the time of transition between acute and chronic phase, similar to what has been observed in SMs [39].

We monitored the activation profiles of CD16^+^ and CD16^−^ NK cells in blood and of the CD16^−^ NK cells in LNs. As shown in Figure 4C and E, the frequencies of Ki-67^+^ and CD69^+^ NK cells were markedly enhanced upon SIVagm infection in blood with a peak on day 11 pi. The activation profiles of CD16^−^ NK cells in blood followed a similar kinetic than that of CD16^+^ NK cells (not shown). The percentage of activated NK cells also highly increased in the LNs (Figure 4D and F).

To evaluate NK cell function, the surface expression of CD107a (a surrogate marker for cytolytic function) and intracellular expression of IFN-γ (cytokine production) were measured (Figure 4G–J). The NK cell cytolytic activity was significantly increased only in LNs (Figure 4G and H) and no significant increase of IFN-γ production was observed in either blood or LNs (Figure 4I and J).

NK activation in blood and LNs was correlated with viral replication (blood CD16^+^Ki-67%: Rs = 0.37, p<0.001; LN CD16^−^Ki-67%: Rs = 0.54, p = 0.002; blood CD16^+^CD69%: Rs = 0.4, p<0.001; LN CD16^−^CD69%: Rs = 0.53, p = 0.002). The NK cell cytolytic activity in LNs was also correlated with viral load (CD16^−^CD107a%: Rs = 0.54, p = 0.003). Thus, in SIVagm primary infection, NK cells were strongly activated and cytolytic NK cells increased in LNs. These increases were positively associated with viremia levels.

### Interferon-α production correlated with NK cell activation

**Figure 5:**
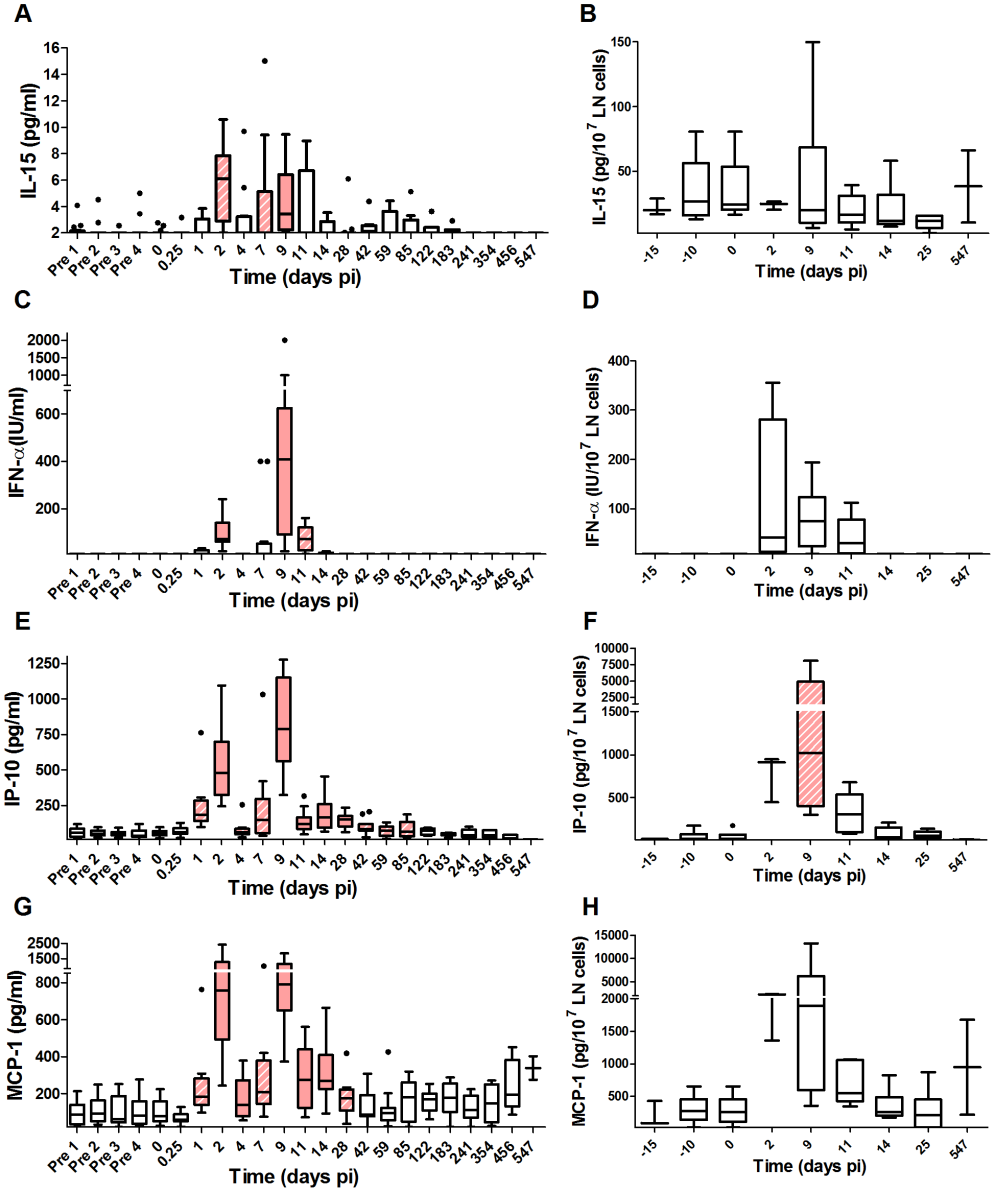
Profiles of early cytokines in plasma and lymph node supernatants upon SIVagm infection. Cytokine concentrations in plasma (n = 14 AGMs) and LN cell supernatants (n = 6 AGMs), as determined by ELISA or by a functional test for IFN-α: (A, B) IL-15, (C, D) IFN-α, (E, F) IP-10 and (G, H) MCP-1. The graphs are listed by order of appearance of the cytokines as described for pathogenic HIV infection [43]. Only cytokines known to be induced very early on in HIV infection are shown. The horizontal line in each box plot represents the median. The bottom and top edges of each box correspond to the 25th and 75th percentiles. Whiskers extend up to adjacent values representing the largest and smallest values that are not outliers. Day zero represents the median of all the time points before infection. Red and hatched boxes indicate statistically significant increases relative to baseline with p<0.001 and 0.001<p<0.05, respectively (Wilcoxon matched-pairs signed rank test). The X axes were placed at the threshold value.

Both NK cell activation and cytotoxic activity are stimulated by IFN-I, which is driven by virus. IL-15 plays a pivotal role in the development, survival and function of NK cells. We quantified IFN-I and IL-15 concentrations in blood and tissues. In line with previous reports for SIVagm infection [36], [38], [42], the IFN-α levels in plasma were transiently increased during primary infection. In addition, we reveal an increase of IL-15 production. The animals displayed two peaks of IFN-α and IL-15 production, on days 2 and 9 pi, day 9 corresponding to the peak of plasma viremia (Figure 5A and C). By day 11 pi, these levels were already decreased and below detection limit after day 14 pi for IFN-α. IFN-α and IL-15 were also measured in LNs *ex vivo* by collection of supernatants from the LN cell preparations (Figure 5B and D). The IFN-α concentrations in these supernatants were increased between days 2 and 11 pi. The limited number of LNs that could be collected did not allow for the same close monitoring frequency as in blood and it is unclear whether two peaks of expression were present in the LN compartment as well.

We found that NK cell activation in blood was correlated with the IFN-α levels (CD69%: Rs = 0.39, p<0.001; Ki-67%: Rs = 0.24, p = 0.008) and the IL-15 levels in plasma (CD69%: Rs = 0.25, p = 0.009; Ki-67%: Rs = 0.28, p = 0.003). At the level of LNs, IFN-α was correlated with NK cell activation (CD69%: Rs = 0.35, p = 0.032; Ki-67%: Rs = 0.41, p = 0.012) and cytotoxicity (CD107a%: Rs = 0.5, p = 0.002), while IL-15 was only correlated with NK cell proliferation (Ki-67%: Rs = 0.43, p = 0.007). The IFN-α levels also correlated with Ki-67^+^ CD4^+^ T cell levels (Rs = 0.3, p = 0.002). Altogether, IFN-α and IL-15 correlated with NK activation and IFN-α with NK cytotoxic activity in LNs.

### Increased cytokines in early but not late acute phase of SIVagm infection

We quantified thirteen additional cytokines in the plasma for the two AGM groups (Figure 5 and S2) to determine the earliest kinetics of cytokines and search for differences with the cytokine storm reported in HIV-1 and SIVmac infections. Cytokines reported to be increased early during HIV-1 infection were selected, such as MCP-1 as well as “innate” cytokines, such as IL-12. The early collection time points were chosen at very short intervals starting at 6 hours pi. Among the 15 cytokines studied in total, 8 were significantly up-regulated and 6 displayed a first peak on day 2 as well as a second increase on day 7 and/or 9 pi: IL-15, IFN-α, IP-10, MCP-1, IFN-γ and IL-18 (Figure 5 and S2). IL-15, IP-10 and MCP-1 are inducible by IFN. Their profiles strongly correlated with IFN-α levels (IL-15: Rs = 0.53, p<0.001; IP-10: Rs = 0.73, p<0.001; MCP-1: Rs = 0.6, p<0.001) (Figure 5). IL-8 was modestly increased at day 9 pi, while IL-12 was up-regulated only later on, on days 14 and 28 pi and even downregulated at time points just after the IFN-α peaks (Figure S2). This might be due to the fact that IL-12 is inhibited by IFN-α [54]. As a matter of fact, previous reports in SIVmac and HIV-1 infection showed that IL-12 levels increase late in primary infection, once the IFN-α levels decrease [43], [55]. Strikingly, in SIV-infected AGMs, most of the other pro- and anti-inflammatory proteins and ISPs measured (IL-6, sTRAIL, TNF-α, IL-17, TGF-β) were not modulated (Figure S2).

We wondered whether the first peak (day 2 pi) is specific for natural hosts. For those cytokines, which showed increases already on day 2 pi in AGMs, we also measured cytokines in two rhesus macaques infected with SIVmac251 (Figure S3). Also in these monkeys, a peak of cytokine production was observed on day 2 pi depending on the cytokine and the animal studied, suggesting that the early peak is not unique to AGMs. Most of studies conducted so far don't include such early time points. However, a similar early induction (day 2–4 pi) of IFN-α and some ISGs has been observed at mucosal sites of orally infected macaques [56]. We had already noticed such an early peak in previous studies [36], [38]. The AGMs were infected here with a purified virus which therefore excludes that the early peak is due to contaminants in the *inoculum*. Finally, the data confirm previous reports showing that IFN-α levels are lower in acutely infected AGMs compared to macaques (Figure S3B) [35], [36], [38].

Altogether, the close monitoring of fifteen soluble factors showed that cytokines, which are known to be produced early during SIVmac infection in macaques or HIV-1 infection in humans, i.e. before, during or shortly after the viral peak, were all induced in SIVagm infection. In contrast, cytokines that are induced late during the acute phase of pathogenic infection were not or only moderately induced in AGMs.

### Administration of high levels of IFN-α during primary infection has no impact on SIVagm infection

We tested whether the lower levels of IFN-α in SIVagm infection might dictate the outcome of infection, in particular with respect to the resolution of the inflammation. We therefore administered high doses of recombinant IFN-α (r-mamu-IFN-α) during the acute phase of SIVagm infection in an attempt to perturb the control of inflammation and abolish its resolution, which would be characterized by uncontrolled expression of ISGs and chronic immune activation.

**Figure 6:**
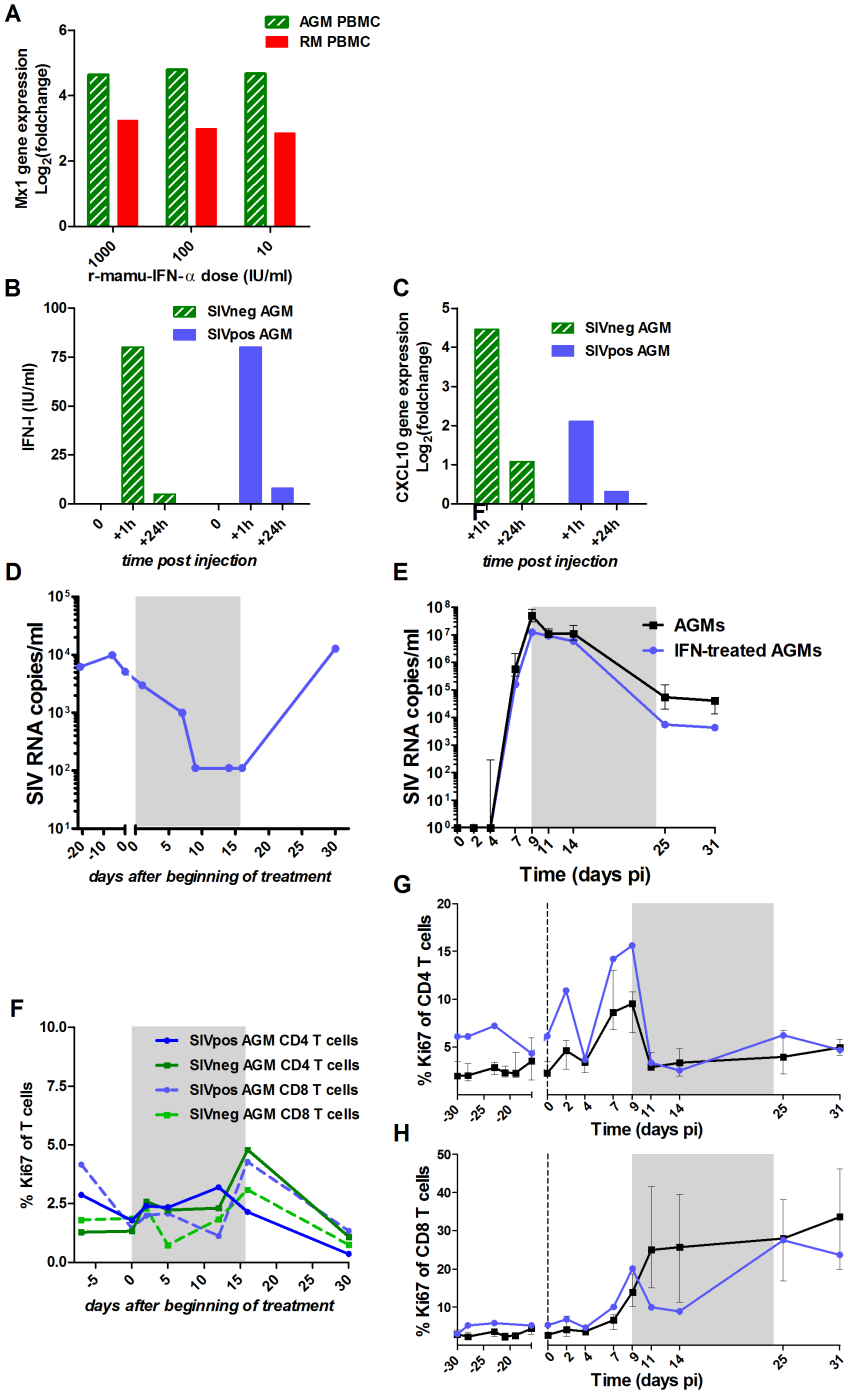
Evaluation of r-mamu-IFN-α efficacy in AGMs. (A) *In vitro* comparison of the induction of ISG expression in AGM (hatched green) *versus* rhesus macaques (red) PBMCs after 18 h stimulation with 10, 100 or 1000 IU/ml of r-mamu-IFN-α (n = 2 AGMs, n = 2 macaques). *Mx1* gene expression values were measured by real-time PCR and are expressed as the Log_2_ (fold change relative to values before treatment). The experiment was done in triplicate. (B) IFN-α detection in the plasma of SIV-negative (hatched green) and SIV-infected (blue) AGMs after a single subcutaneous injection of 5×10^5^ IU of r-mamu-IFN-α. IFN-α was rapidly detectable in blood (1 h), but decreased after 24 h. (C) Efficient induction of ISG (*CXCL10*, *IP-10*) in the PBMC of SIV-negative (hatched green) and SIV-infected (blue) AGMs (chronic phase) after a single subcutaneous injection of 5×10^5^ IU of r-mamu-IFN-α. Gene expression was measured by real-time PCR and expressed as the Log_2_ (fold change relative to values before treatment). (D) Viral load in a chronically SIV-infected AGM treated during 16 days with r-mamu-IFN-α as described in results. (E) Impact of the r-mamu-IFN-α treatment during acute infection on viral load. SIVagm-infected untreated AGMs are shown in black and IFN-α-treated in blue. Plasma viral load are reported as log_10_ (viral RNA copies)/ml of plasma. (F) No major effect of the two weeks IFN-α treatment on T cell proliferation in an uninfected AGM and in a chronically infected AGM (same AGM as in D) as measured by flow cytometry. (G and H) No effect of the treatment during the acute phase on T cell proliferation. T cell proliferations are shown as percentage of Ki-67^+^ cells among blood (G) CD4^+^ and (H) CD8^+^ T cells. SIVagm-infected untreated AGMs are shown in black and IFN-α-treated in blue. The grey area indicates the period of treatment. The medians of treated animals were inside the interquartile range of the control untreated animals and were thus considered not different.

The r-mamu-IFN-α used for the *in vivo* treatment was first tested for its efficacy on AGM cells *in vitro* and *in vivo* (Figure 6A–D and F). The same cytokine has been previously used in SMs without inducing any anti-IFN-α antibodies [57]. AGM PBMCs exposed to r-mamu-IFN-α *in vitro* up-regulated the expression of ISGs, such as *Mx1* or *IP-10*, to similar levels as in macaque PBMCs (Figure 6A). Low doses of the r-mamu-IFN-α were already highly efficient for ISG induction, in line with previous data [36]. After a single *in vivo* injection of 5×10^5^ IU of r-mamu-IFN-α, high levels of IFN-α were observed in plasma 1 hour post-treatment (Figure 6B), leading to strong up-regulation of ISGs, such as *IP-10* (Figure 6C). Since the half-life of a similar human recombinant IFN-α has been estimated at 2–5 h in AGMs *in vivo* [58], this could explain why the levels of IFN-α and *IP-10* mRNA were already low at 24 h after administration despite the r-mamu-IFN-α being an IgG fusion protein [57], [59]. In order to maintain robust levels of IFN-α and constantly high expression of ISGs, we injected r-mamu-IFN-α daily with a 10% increment every 2 days for 16 days. The safety and efficacy of such treatment were verified on a SIV-chronically-infected AGM. The latter displayed a 2 log_10_ decrease of the chronic viral load during the treatment (Figure 6D), but no major difference in T cell activation (Figure 6F), similar to data reported for chronically SIV-infected SMs [57]. Anti-IFN-α antibodies were not detected at any time point during or after treatment (data not shown).

**Figure 7:**
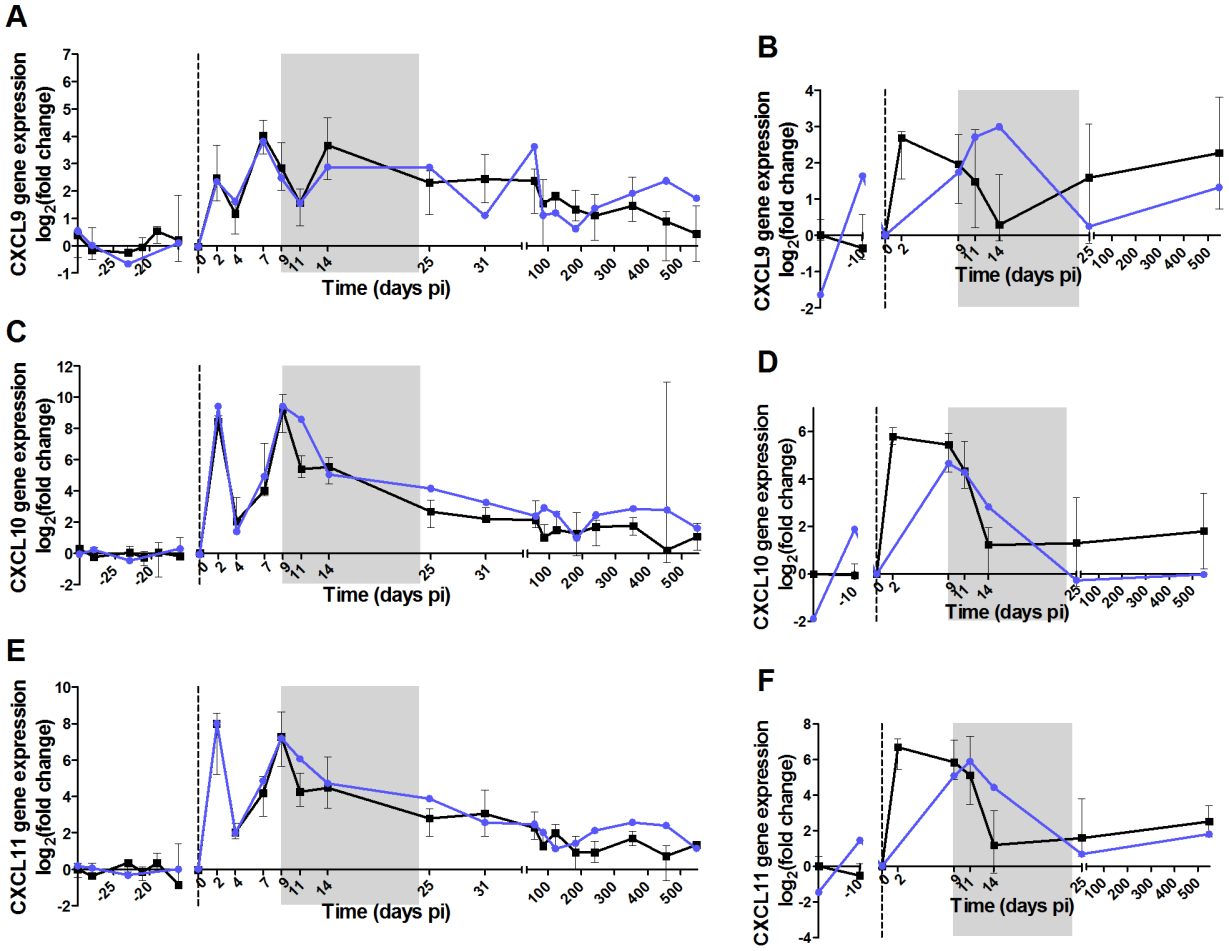
High dose of IFN-α in the acute phase of infection does not induce persistent ISG expression in AGMs. The 6 panels show the expression levels in PBMCs and LN cells of 3 ISGs: (A,B) *CXCL9*, (C,D) *CXCL10* (*IP-10*) and (E,F) *CXCL11*. Gene expression was measured by real-time PCR and expressed as the Log_2_ (fold change relative to values before treatment). IFN-α treated AGMs are in blue and untreated in black. The grey area indicates the period of treatment. The medians of treated animals were inside the interquartile range of the untreated animals and were thus considered not different.

Since r-mamu-IFN-α was efficient in cells from uninfected and chronically infected AGMs, we then tested whether such treatment would affect the resolution of immune activation during primary infection. The treatment was started on day 9 pi because after day 9 pi, endogenous IFN-α levels started to decrease, concomitantly with the diminution of other cytokines and ISPs such as IP-10 and MCP-1 and the decrease of activated NK cells and Ki-67^+^ CD4^+^ T cells. Also, based on data from the literature, we could not exclude that an initial inflammation during the first week of infection is necessary to establish infection. Finally, we did not want to interfere with the efficacy of the initial viral replication. Two AGMs were injected daily with r-mamu-IFN-α between day 9 and 24 pi. The virological and immunological profiles in the IFN-α-treated AGMs were compared to those of the 6 infected but untreated AGM (Figures 6E, 6G, 6H, 7, S3 and S4).

The administration of r-mamu-IFN-α during the acute phase of infection had no major effect on viral load (Figure 6E), even if a slight but not significant decrease was observed as compared to untreated animals at the first time points after treatment. Body temperatures were elevated during the treatment period with IFN-α (Figure S5). The expression of the ISGs *CXCL9*, *IP-10* and *CXCL11* were however comparable between treated and untreated AGMs in both PBMCs and LN cells (Figure 7). The treatment also did not result in a persistent T cell activation (Figure 6G and H) or CD4^+^ T cell loss over time (data not shown). As IFN-α is known to exert direct and indirect effects on innate immune cells, we also investigated whether in the absence of a change in disease outcome, the IFN-α treatment would still have had an impact on NK cells, mDCs and pDCs (Figure S4). The administration of r-mamu-IFN-α in the context of the SIV primary infection did not affect their frequencies and did not induce an increase of maturation or activation of these innate immune cells.

In summary, in spite of daily administration of high doses of IFN-α post peak of SIV replication, AGMs were still able to resolve inflammation and immune activation.

## Discussion

We aimed to study if the lower levels of IFN-α described during SIVagm infection as compared to SIVmac infections matter in the resolution of the inflammation in AGMs. The early host immune responses are an essential factor in determining the subsequent clinical course of disease. In mice, an early innate alteration significantly compromises the following immune responses and the host's ability to counteract the virus/parasite spread [60], [61]. We tested here whether by artificially increasing IFN-α related inflammation during the acute phase of SIVagm infection, one can overcome the intrinsic control of immune activation in this natural host. In order to study the control of immune activation and not interfere with the establishment of viral infection, the treatment was administered between the plasma viral peak and the end of the acute phase of infection. Surprisingly, the treatment did not affect viral dynamics, control of inflammation or T cell activation. This is not due to a lack of sensitivity of AGM cells to the recombinant IFN-α used here. Indeed, the r-mamu-IFN-α molecule was functional *in vitro* and *in vivo* in healthy and chronically-infected AGMs. Moreover, when administered during the chronic phase of infection, our results paralleled those described in chronically-infected SMs treated with the same molecule, namely a reduction in viral load and increase in ISG expression in the absence of major increases of T cell activation [57]. Although the number of animals was low, the analyses show that r-mamu-IFN-α was fully active on AGM cells.

It is possible that the lack of changes after IFN-α treatment during primary SIVagm infection was due to tolerance to the injected IFN-α. In SMs chronically infected with SIVsmm and treated with IFN-α, the effect of IFN-α was transient likely due to the induction of tolerance to such treatment as also reported in humans [62], [63]. Here, the treatment was short, but refractoriness could have been induced by the previous response to endogenous high levels of IFN-α. Of note, we treated the animals starting from day 9 pi, corresponding to the peak of endogenous IFN-α production. Had we started the treatment on the day of infection, we cannot exclude that we might have seen an effect on ISGs or viral load. However, such protocol might have lowered the initial viral replication, which was in opposition with the aim of our study. Altogether, administration of IFN-α in the mid and late part of the acute phase did not change the outcome, suggesting that the resolution of inflammation in AGMs is not due to a difference in the levels of IFN-α production during primary infection. It has been suggested that the combination of antiretroviral therapy and interferon given during acute HIV infection may potentiate both innate and adaptive immune responses against HIV replication and/or reservoir levels [64]. Our study shows that in primary infection, IFN-α, when administered after the peak of viremia, does not affect viral replication or innate responses. It reduces viral load during chronic phase. Whether this is true for pathogenic infection, remains to be determined. However, the timing is very important and should be considered when such treatment is envisaged.

We previously showed that during the acute phase of SIV infection the levels of ISGs, such as of IP-10, strictly correlate with IFN-α levels [36]. Here, treatment with high doses of IFN-α did not lead to sustained ISG expression. This suggests that, at the transition of acute to chronic phase other factors than IFN-α predominantly drive ISG expressions in macaques and humans. Elevated expression levels of ISGs in chronic infection are associated with uncontrolled viremia and disease progression [23], [65]. It could be that not the IFN-I production, but constant ISG expression are deleterious for the host. IP-10 has been reported to be an excellent marker of inflammation and disease progression [4], [65]–[68]. IP-10 is inducible not only by IFN-I and IFN-γ, but also by other pro-inflammatory cytokines (TNF-α, IL-1β, IL-18) [69]–[71]. Hence, it is possible that IFN-α alone is not sufficient, but that a combination with other factors, TNF-α for example, is also required to induce ISG expression. Moreover, even though ISGs are IFN-inducible, some were shown to be directly up-regulated through recognition of viral or bacterial products by pattern-recognition receptors in an IFN-independent manner [72]–[75]. Finally, an expansion of the enteric virome and microbial translocation are observed in chronic HIV-1 and SIVmac infections [76], [77]. It could thus explain why macaques and humans maintain ISG expression but not AGMs who do not display microbial translocation or virome expansion. Other or additional factors might also play a role in the maintenance of ISG expressions during HIV-1/SIVmac infections. For instance, distinct IFN-α subtypes differently induce ISG expressions *in vivo*, while here, only IFN-α2, which is considered the most abundant in viral infections, was used [69], [78]–[80].

Altogether, our study indicates that the non-pathogenic outcome of SIVagm infection is not due to differences in IFN-α levels between AGM and macaques or humans. It does not exclude that the difference in outcome is related to different levels of ISG expression. It indicates however, that the mechanisms, which maintain high levels of ISG expression, are due to other or additional factors than IFN-α.

Several studies have debated whether natural hosts display lower or similar immune activation levels during primary infection as compared to pathogenic infections. Some studies have reported weaker levels of T cell activation and cytokine concentrations during the acute phase of SIVagm or SIVsmm infection, whereas in other studies the levels were equivalent to those observed in SIVmac-infected macaques [30], [36], [41], [81]. We performed a detailed follow-up of T cell activation and, to attempt to reconcile the discrepant cytokine profiles, we deciphered here the early production of cytokines in AGM. We included in the study the cytokines known to be induced very early during pathogenic infection, such as IL-15 [19], [43]. Of note, the acute phase of SIVagm infection resulted in significant increases of early cytokines, including IL-15, IP-10 and MCP-1, similar to pathogenic infection. However, salient differences were observed for cytokines known to be produced in later stages of the acute phase of HIV-1/SIVmac infections. They were either not or only weakly induced in AGMs. In particular, IL-6 and TNF-α were not up-regulated (Figure S2). We hypothesize that the early cytokines, which are produced in AGMs during the first two weeks pi, confer a benefit to both the virus and the host. It would be beneficial to the virus as inflammation attracts target cells to the sites of infection. For the host, the induction of early innate responses (restriction factors, NK cells, mDCs), would allow the development of antiviral innate and adaptive responses for partial control of viral replication. AGMs might have found a way to allow early inflammation resulting in productive infection while blocking the cytokine storm that takes place following the viral peak. This would avoid the sustained inflammatory environment.

The dual pattern of cytokines that we observed might be explained by a differential susceptibility to activation by the innate cells. While pDCs show a normal sensing of SIVagm [36], [37], [40] leading to the production of IFN-α, other cells, for instance myeloid cells, such as mDC and macrophages, might not produce any cytokine. Indeed, a recent study reported that in contrast to SIVmac and HIV-1 infections, mDCs mature but do not show spontaneous production of pro-inflammatory cytokines such as TNF-α in primary SIVagm infection [48].

To understand what might be the key events of the innate response in natural hosts that allows them to maintain the inflammation under control, we investigated the effect of SIVagm infection in AGM on innate immune cell compartments, in particular pDCs, mDCs and NK cells. Little is known about those decisive early cellular responses in AGMs, in particular regarding pDC maturation and NK cell activation. In addition, for the first time the activation profiles of these three types of innate cells were analyzed concomitantly in the same animals. We analyzed the maturation and homing patterns of two sub-populations of mDCs, the CD16^−^ and CD16^+^ subsets. These correspond to two major subsets of mDCs in humans [82], [83]. The CD16^+^ mDCs displayed higher levels of activation and maturation than the CD16^−^ subpopulation. Whether these cells play a distinct role in T cell activation or tolerance is unclear. PDCs displayed lower levels of maturation than mDCs. IFN-α production by pDCs is associated with their maturation stage. It has been shown that HIV skews pDCs toward a partially matured and persistently IFN-α-secreting phenotype which allows their survival [84]. Eventually, the partial maturation of pDCs in AGMs might be associated with their capacity of efficient IFN-α production during the acute phase of SIVagm infection. Egress of pDC precursors from bone marrow could then account for the return of IFN-α levels to baseline [85]. This is supported by the decrease of HLA-DR expression on the pDC's surface. On the contrary, a preferential maturation process at the expense of cytokine secretion might be occurring at the level of mDCs in AGMs, especially in presence of IFN-I, since IFN-I induces mDC maturation rather than cytokine secretion [83].

We also analyzed NK cells for the first time in the context of SIVagm infection. We observed a strong increase in proliferation and activation of NK cells during the acute phase of SIVagm infection. Our data support the observations reported in SMs on earlier and stronger NK cell responses than in SIVmac-infected macaques [39]. The rapid and strong increase in NK cell proliferation in AGMs might be a direct consequence of the early and robust production of IL-15 and IFN-α during primary SIVagm infection. No production of IFN-γ by NK cells was observed, while NK cell cytotoxicity was induced. It has been shown that IFN-α and IL-15 promote NK cell proliferation and survival, while IFN-α is able to increase NK cell cytotoxicity, and IL-12 to augment the secretion of IFN-γ [86]. This is in accordance with the fact that modest levels of IL-12 and high levels of IFN-α were detected, playing thus a putative role in establishing such protective NK cell responses. It was surprising to detect increases in NK cytotoxic function in LNs. One hallmark of SIV infection in natural hosts is the high viral load in blood and intestinal tissues, but low viral burden in LNs in the chronic phase of infection [29], [87]–[90]. It has been suggested for SMs that the rapid and dramatic control of viral replication in LNs is associated with CD8^+^ T cell responses [89]. However, it is tempting to speculate that at least in the early stage of SIVagm infection in AGMs, NK cells could significantly contribute to the control of viral replication in LNs which in consequence could contribute to limit immune activation [91].

Altogether, our study provides evidence that the control of immune activation in SIVagm infection is not a consequence of lower levels of IFN-α production. We show that AGMs mount strong early innate immune responses as exemplified by the significant NK cell activation and production of early cytokines, such as IL-15 and MCP-1. Our study indicates that the sustained ISG production in HIV/SIVmac infections is likely driven by additional or factors other than IFN-α, among which could be elevated pro-inflammatory cytokine levels, enteric virome expansion and microbial translocation. The data also suggest that mechanisms controlling inflammation are in place before the transition of the acute to the chronic phase, thus earlier than previously considered. Whether this is due to the establishment of inhibitory or tolerance mechanisms after the viral peak, or to a distinct susceptibility to infection or immune activation by specific immune cell subsets, needs to be further investigated.

## Materials and Methods

### Ethics statement

Animals were housed in the facilities of the CEA (“Commissariat à l'Energie Atomique”, Fontenay-aux-Roses, France) and Institut Pasteur (Paris, France) (CEA permit number: A 92-032-02, Institut Pasteur permit number: A 78-100-3). All experimental procedures were conducted in the CEA animal facility and in strict accordance with the international European guidelines 2010/63/UE about protection of animals used for experimentation and other scientific purposes (French decree 2013-118) and with the recommendations of the Weatherall report. The monitoring of the animals was under the supervision of the veterinarians in charge of the animal facilities. All efforts were made to minimize suffering, including efforts to improve housing conditions and to provide enrichment opportunities (e.g., 12∶12 light dark schedule, provision of monkey biscuits supplemented with fresh fruit and constant water access, objects to manipulate, interaction with caregivers and research staff). All procedures were performed under anesthesia using 10 mg of ketamine per kg body weight. For deeper anesthesia required for lymph node removal a mixture of ketamine and xylazine was used. Paracetamol was given after the procedure. Euthanasia was performed prior to the development of any symptoms of disease (e.g., for macaques when the biological markers indicated progression towards disease, such as significant CD4^+^ T cell decline and increases of viremia). Euthanasia was done by IV injection of a lethal dose of pentobarbital. The CEA is in compliance with Standards for Human Care and Use of Laboratory of the Office for Laboratory Animal Welfare (OLAW, USA) under OLAW Assurance number #A5826-01. Animal experimental protocols were approved by the Ethical Committee of Animal Experimentation (CETEA-DSV, IDF, France) (Notification number: 10-051b).

### Animals, treatments and sample collections

Eighteen Caribbean-origin African green monkeys (*Chlorocebus sabaeus*) and two Chinese rhesus macaques (*Macaca mulatta*) were used in the study. AGMs were infected by intravenous inoculation with 250 TCID_50_ of purified SIVagm.sab92018, and macaques with 5000 AID_50_ of SIVmac251, as previously described [90]. SIVagm.sab92018 has been purified by sucrose density gradient centrifugation and on Vivaspin 20 columns (Vivaproducts). Neither IFN-α nor endotoxin (LAL QCL-1000 Kit, Lonza) were detected in the two viral stocks. Four AGMs were treated with the r-mamu IFN-α-IgFc by subcutaneous injection (Resource for NHP Immune Reagents, Emory University, Atlanta, GA): 2 were used for the establishment of the treatment protocol and control of its efficiency in AGM, and 2 were treated during the acute phase of infection. When daily injections of 5×10^5^ IU for over a period of 16 days were performed, the dose was increased by 10% every second day.

Whole blood was collected from all AGMs. For the initial groups of monkeys, baseline blood collections were performed at 4 to 6 time points before infection (days −30, −28, −23, −21, −19 and −16) to mimic the sampling of the acute phase and measure any difference linked to the sampling. No variation due to the sampling was observed. Blood was then collected during primary infection (on days 2, 4, 7, 9, 11, 14 and 25) and during the chronic phase (days 31, 59, 85, 122, 183, 241, 354, 456, 547 or euthanasia). AGM peripheral LNs were obtained by excision before infection (days −15 and/or −10) and after infection at the following days: 2 (3 AGMs), 7 (1 AGM), 9 (8 AGMs), 11 (7 AGMs), 14 (8 AGMs), 25 (7 AGMs) and 547 pi or euthanasia (5 AGM). In a second group of 8 AGMs, blood was collected at 3 to 4 time points before infection (days −40, −30, −20, −10), at very early time points during primary infection (6 hours post-infection and at days 1, 2, 4, 7, 9, 11, 14, 28 pi) and during the chronic phase (days 42 and 63 pi).

### Flow cytometry

Panels of fluorochrome-labeled monoclonal antibodies (mAbs) that have been shown to be cross-reactive with AGMs, were used to label fresh whole blood and LN cells and were purchased from BD Biosciences unless otherwise stated: CD3 (SP34-2), CD4 (L200), CD8 (Sk1), CD20 (2H7, ebioscience), HLA-DR (L243), CD16 (3G8), NKG2A (Z199, Coulter), CD107a (H4A3), CXCR3 (1C6), CD69 (FN50, ebioscience), CD123 (7G3), BDCA-2 (AC144, Miltenyi), CD86 (FUN-1), CD40 (HB14, Caltag), CCR7 (3D12, ebioscience), CD11c (S-HCL-3), CD80 (L307.4), IFN-γ (45-15, Miltenyi), CD45 (D058-1283), Ki-67 (MIB-1, Dako) as well as isotype controls. FcR Blocking Reagent (Miltenyi) was used to block unwanted binding of antibodies and increase the staining specificity of cell surface antigens. For detection of IFN-γ and CD107a, cells were pre-incubated for 4 hours with brefeldin A and monensin at 37°C prior to labeling with surface-binding antibodies and then fixed and permeabilized prior to incubation with IFN-γ antibody. Cells were run on a BD LSR-II flow cytometer system, collected with BD FACS Diva 6.0 software, and analyzed with FlowJo 8.8.7 (TreeStar).

### Cytokine quantifications

Cytokines were measured in plasma and LN cell supernatants. LN cell supernatant consists of the medium in which the biopsy was collected and kept for 2–3 hours at 4°C. Cells were prepared by homogenization in the same medium and the supernatant was collected after centrifugation. Titers of bioactive IFN-α were determined as previously described [38]. The same test was used to search for plasma IFN-α antibodies that might have developed in response to the treatment. The other cytokines were quantified using the following ELISA kits: MONKEY IFN-gamma, IL-6, IL-8, IL-10, IL-12/23p40, TNF-alpha (U-Cytech), Human IL-15, CXCL10/IP-10, CCL2/MCP-1, TRAIL/TNFSF10 Quantikine Kits (R&D), Human IL-17A Ready-SET-Go (eBiosciences), Human IL-18 Kit (MBL), Simian IFN-beta Kit (USCN), TGF-β1 Multispecies Kit (Invitrogen). To verify the cross-reactivity of the antibodies used in the ELISA kit for cytokines that have never been tested on AGM [30], [36], AGM PBMCs were stimulated *in vitro* and cytokines were measured in the supernatants (data not shown).

### Quantification of viral RNA and of cellular mRNA

Plasma viral load was determined by real-time PCR [90]. Quantification of *ISG* transcripts was performed by real-time RT-PCR in triplicate using Taqman gene expression assays (Life technologies). The expression of each gene was normalized against that of *18S* rRNA [30], [36].

### Statistical analyses

To characterize each marker's progression (figures 2–4), a linear mixed effect model was used to account for multiple measurements within each AGM. Firstly, we graphically assessed that the marker's distribution was Gaussian; if not, a logarithmic transformation was used. Secondly, a LOWESS (locally weighted scatterplot smoothing) curve was used to assess whether the marker's trajectory looked linear or piecewise linear. Based on these trajectories, we introduced or not slopes. We indicated at which time-point the change of slope occurred. Finally, a mixed effect linear or piecewise-linear model was applied. When two slopes were introduced into the model, the difference between the two slopes was tested using Wald's test. The Wilcoxon matched-pairs signed rank test was used to evaluate whether there was a statistically significant difference in the level of one given marker at a given time point following inoculation when compared to the baseline medians (day 0), using Prism (GraphPad). Baseline medians in blood consisted of 3 to 6 pre-infection values per animal for the flow cytometry and gene expression analysis, and 4 total pre-infection values per animal for the plasma cytokines study. In LNs, it consisted of 1 to 2 pre-infection values per animal for all the measurements. Finally, the Spearman rank test was used to assess the correlation between 2 continuous variables.

## Supporting Information

Figure S1 **Flow-cytometric gating strategy.** Flow-cytometric gating strategy used to identify (A) pDCs and mDCs, (B) CD16^+/−^ NK cells in blood and (C) CD16^−^ NK cells in LNs. Representative flow-cytometric analysis with whole blood (A, B) and LN cells (C) from a healthy AGM.
(TIF)

Figure S2 **Levels of soluble factors in plasma during SIVagm infection.** Plasma levels of 11 additional cytokines were evaluated. The number of animals are indicated in brackets after each cytokine: (A) IFN-β (n = 6), (B) IFN-γ (n = 14), (C) IL-18 (n = 6), (D) IL-8 (n = 8), (E) sTRAIL (n = 8), (F) IL-6 (n = 8), (G) IL-12p40 (n = 14), (H) TGF-β (n = 8), (I) IL-10 (n = 8) were measured by ELISA. The results for IL-17 and TNF-α ELISA are not shown since the levels were undetectable in AGM plasma. Red hatched boxes indicate statistically significant increases relative to baseline with p<0.05. Blue boxes indicate a decrease (p<0.05) (Wilcoxon matched-pairs signed rank test). The X axes were placed at the threshold value.
(TIFF)

Figure S3 **Comparison of the cytokine levels and kinetics in treated and untreated AGMs with those of rhesus macaques.** Plasma levels of cytokines in 14 AGMs infected with SIVagm.sab92018 (black), the 2 IFN-α treated AGMs (blue) and 2 rhesus macaques infected with SIVmac251 (RM) (red). The levels of 4 early cytokines (A) IL-15, (B) IFN-α, (C) IP-10, (D) MCP-1 and 2 cytokines reported to appear later in SIVmac/HIV-1 primary infection (E) IFN-γ and (F) IL-18 (n = 6 AGMs) were determined. Data are presented as medians and interquartile ranges for untreated AGMs and as median only for the 2 treated AGMs and the 2 RMs. Day zero represents the median of all the time points before infection. All the AGMs and RMs were infected on day zero. The grey area indicates the period of IFN-α treatment.
(TIF)

Figure S4 **High dose of recombinant IFN-α injection during primary SIVagm infection does not affect innate immune cells.** Analysis of the effect of high dose IFN-α injection *in vivo* on NK cells (A, B) frequencies and (C, D) activation (Ki-67%), on pDCs (E, F) frequencies and (G, H) maturation (CD86 mfi) and on mDCs (I, J) frequencies and (K, L) maturation (CD80 mfi), in blood (left panels) and LNs (right panels). Data are presented as medians and interquartile ranges for untreated animals (black) and median only for treated animals (blue). Day zero represents the median of all the time points before infection. The grey area indicates the period of treatment. The medians of treated animals were inside the interquartile range of the control untreated animals and were thus considered not different.
(TIF)

Figure S5 **Effect of injection with high doses of recombinant IFN-α during primary SIVagm infection on body temperature.** Body temperature changes as compared to temperature either before infection (BI) or before treatment (day 9 p.i.) for the 2 treated AGMs (blue) and 6 untreated AGMs (black). The temperature values after infection but before IFN-α-treatment correspond to time points between days 2 and 9 pi and are shown on the left. In the IFN-α-treated animals (between days 11 and 25 pi) on the right, the changes are indicated relative to the time point before treatment initiation (day 9 pi) and compared to the body temperature of the 6 untreated AGMs during the same time period. The median is indicated.
(TIF)

## References

[1] Giorgi JV, Hultin LE, McKeating JA, Johnson TD, Owens B, et al (1999) Shorter survival in advanced human immunodeficiency virus type 1 infection is more closely associated with T lymphocyte activation than with plasma virus burden or virus chemokine coreceptor usage. J Infect Dis 179: 859–870. 10068581 10.1086/314660

[2] Kuller LH, Tracy R, Belloso W, Wit SD, Drummond F, et al (2008) Inflammatory and Coagulation Biomarkers and Mortality in Patients with HIV Infection. PLoS Med 5: e203. 18942885 10.1371/journal.pmed.0050203 PMC2570418

[3] Tien PC, Choi AI, Zolopa AR, Benson C, Tracy R, et al (2010) Inflammation and Mortality in HIV-Infected Adults: Analysis of the FRAM Study Cohort. JAIDS Journal of Acquired Immune Deficiency Syndromes 55: 316–322 310.1097/QAI.1090b1013e3181e66216. 20581689 10.1097/QAI.0b013e3181e66216 PMC2955817

[4] Liovat AS, Rey-Cuille MA, Lecuroux C, Jacquelin B, Girault I, et al (2012) Acute plasma biomarkers of T cell activation set-point levels and of disease progression in HIV-1 infection. PLoS ONE 7: e46143. 23056251 10.1371/journal.pone.0046143 PMC3462744

[5] Roberts L, Passmore JA, Williamson C, Little F, Bebell LM, et al (2010) Plasma cytokine levels during acute HIV-1 infection predict HIV disease progression. Aids 24: 819–831. 20224308 10.1097/QAD.0b013e3283367836 PMC3001189

[6] Reschner A, Hubert P, Delvenne P, Boniver J, Jacobs N (2008) Innate lymphocyte and dendritic cell cross-talk: a key factor in the regulation of the immune response. Clinical & Experimental Immunology 152: 219–226. 18336590 10.1111/j.1365-2249.2008.03624.x PMC2384094

[7] Frasca L, Lande R (2011) Overlapping, additive and counterregulatory effects of type II and I interferons on myeloid dendritic cell functions. ScientificWorldJournal 11: 2071–2090. 22125457 10.1100/2011/873895 PMC3221594

[8] Beignon AS, McKenna K, Skoberne M, Manches O, DaSilva I, et al (2005) Endocytosis of HIV-1 activates plasmacytoid dendritic cells via Toll-like receptor-viral RNA interactions. J Clin Invest 115: 3265–3275. 16224540 10.1172/JCI26032 PMC1253628

[9] Fonteneau J-F, Larsson M, Beignon A-S, McKenna K, Dasilva I, et al (2004) Human Immunodeficiency Virus Type 1 Activates Plasmacytoid Dendritic Cells and Concomitantly Induces the Bystander Maturation of Myeloid Dendritic Cells. J Virol 78: 5223–5232. 15113904 10.1128/JVI.78.10.5223-5232.2004 PMC400371

[10] Muller-Trutwin M, Hosmalin A (2005) Role for plasmacytoid dendritic cells in anti-HIV innate immunity. Immunol Cell Biol 83: 578–583. 16174110 10.1111/j.1440-1711.2005.01394.x

[11] Kwa S, Kannanganat S, Nigam P, Siddiqui M, Shetty RD, et al (2011) Plasmacytoid dendritic cells are recruited to the colorectum and contribute to immune activation during pathogenic SIV infection in rhesus macaques. Blood 118: 2763–2773. 21693759 10.1182/blood-2011-02-339515 PMC3172794

[12] Nascimbeni M, Perie L, Chorro L, Diocou S, Kreitmann L, et al (2009) Plasmacytoid dendritic cells accumulate in spleens from chronically HIV-infected patients but barely participate in interferon-alpha expression. Blood 113: 6112–6119. 19366987 10.1182/blood-2008-07-170803

[13] Brown KN, Wijewardana V, Liu X, Barratt-Boyes SM (2009) Rapid influx and death of plasmacytoid dendritic cells in lymph nodes mediate depletion in acute simian immunodeficiency virus infection. PLoS Pathog 5: e1000413. 19424421 10.1371/journal.ppat.1000413 PMC2671605

[14] Lehmann C, Lafferty M, Garzino-Demo A, Jung N, Hartmann P, et al (2010) Plasmacytoid dendritic cells accumulate and secrete interferon alpha in lymph nodes of HIV-1 patients. PLoS One 5: e11110. 20559432 10.1371/journal.pone.0011110 PMC2885422

[15] Reeves RK, Evans TI, Gillis J, Wong FE, Kang G, et al (2012) SIV Infection Induces Accumulation of Plasmacytoid Dendritic Cells in the Gut Mucosa. Journal of Infectious Diseases 206: 1462–1468. 22711907 10.1093/infdis/jis408 PMC3529602

[16] Conry SJ, Milkovich KA, Yonkers NL, Rodriguez B, Bernstein HB, et al (2009) Impaired plasmacytoid dendritic cell (PDC)-NK cell activity in viremic human immunodeficiency virus infection attributable to impairments in both PDC and NK cell function. J Virol 83: 11175–11187. 19692459 10.1128/JVI.00753-09 PMC2772797

[17] Reitano KN, Kottilil S, Gille CM, Zhang X, Yan M, et al (2009) Defective plasmacytoid dendritic cell-NK cell cross-talk in HIV infection. AIDS Res Hum Retroviruses 25: 1029–1037. 19795986 10.1089/aid.2008.0311 PMC2828160

[18] Audige A, Urosevic M, Schlaepfer E, Walker R, Powell D, et al (2006) Anti-HIV State but Not Apoptosis Depends on IFN Signature in CD4+ T Cells. J Immunol 177: 6227–6237. 17056552 10.4049/jimmunol.177.9.6227

[19] Fenton-May AE, Dibben O, Emmerich T, Ding H, Pfafferott K, et al (2013) Relative resistance of HIV-1 founder viruses to control by interferon-alpha. Retrovirology 10: 146. 24299076 10.1186/1742-4690-10-146 PMC3907080

[20] Hosmalin A, Lebon P (2006) Type I interferon production in HIV-infected patients. J Leukoc Biol 80: 984–993. 16916960 10.1189/jlb.0306154

[21] Bosinger SE, Hosiawa KA, Cameron MJ, Persad D, Ran L, et al (2004) Gene expression profiling of host response in models of acute HIV infection. J Immunol 173: 6858–6863. 15557180 10.4049/jimmunol.173.11.6858

[22] Hyrcza MD, Kovacs C, Loutfy M, Halpenny R, Heisler L, et al (2007) Distinct transcriptional profiles in ex vivo CD4+ and CD8+ T cells are established early in human immunodeficiency virus type 1 infection and are characterized by a chronic interferon response as well as extensive transcriptional changes in CD8+ T cells. J Virol 81: 3477–3486. 17251300 10.1128/JVI.01552-06 PMC1866039

[23] Sedaghat AR, German J, Teslovich TM, Cofrancesco J Jr, Jie CC, et al (2008) Chronic CD4+ T-cell activation and depletion in human immunodeficiency virus type 1 infection: type I interferon-mediated disruption of T-cell dynamics. J Virol 82: 1870–1883. 18077723 10.1128/JVI.02228-07 PMC2258719

[24] Li Q, Estes JD, Schlievert PM, Duan L, Brosnahan AJ, et al (2009) Glycerol monolaurate prevents mucosal SIV transmission. Nature 458: 1034–1038. 19262509 10.1038/nature07831 PMC2785041

[25] Herbeuval J-P, Shearer GM (2007) HIV-1 immunopathogenesis: How good interferon turns bad. Clinical Immunology 123: 121. 17112786 10.1016/j.clim.2006.09.016 PMC1930161

[26] Keir ME, Rosenberg MG, Sandberg JK, Jordan KA, Wiznia A, et al (2002) Generation of CD3+CD8low Thymocytes in the HIV Type 1-Infected Thymus. J Immunol 169: 2788–2796. 12193754 10.4049/jimmunol.169.5.2788

[27] Liovat AS, Jacquelin B, Ploquin MJ, Barre-Sinoussi F, Muller-Trutwin MC (2009) African non human primates infected by SIV - why don't they get sick? Lessons from studies on the early phase of non-pathogenic SIV infection. Curr HIV Res 7: 39–50. 19149553 10.2174/157016209787048546

[28] Sodora DL, Allan JS, Apetrei C, Brenchley JM, Douek DC, et al (2009) Toward an AIDS vaccine: lessons from natural simian immunodeficiency virus infections of African nonhuman primate hosts. Nat Med 15: 861–865. 19661993 10.1038/nm.2013 PMC2782707

[29] Gueye A, Diop OM, Ploquin MJ, Kornfeld C, Faye A, et al (2004) Viral load in tissues during the early and chronic phase of non-pathogenic SIVagm infection. J Med Primatol 33: 83–97. 15061721 10.1111/j.1600-0684.2004.00057.x

[30] Kornfeld C, Ploquin MJ, Pandrea I, Faye A, Onanga R, et al (2005) Antiinflammatory profiles during primary SIV infection in African green monkeys are associated with protection against AIDS. J Clin Invest 115: 1082–1091. 15761496 10.1172/JCI23006 PMC1062895

[31] Chakrabarti LA, Lewin SR, Zhang L, Gettie A, Luckay A, et al (2000) Normal T-cell turnover in sooty mangabeys harboring active simian immunodeficiency virus infection. J Virol 74: 1209–1223. 10627531 10.1128/jvi.74.3.1209-1223.2000 PMC111455

[32] Pandrea I, Cornell E, Wilson C, Ribeiro RM, Ma D, et al (2012) Coagulation biomarkers predict disease progression in SIV-infected nonhuman primates. Blood 120: 1357–1366. 22653975 10.1182/blood-2012-03-414706 PMC3423778

[33] Bosinger SE, Johnson ZP, Folkner KA, Patel N, Hashempour T, et al (2013) Intact Type I Interferon Production and IRF7 Function in Sooty Mangabeys. PLoS Pathog 9: e1003597. 24009514 10.1371/journal.ppat.1003597 PMC3757038

[34] Bosinger SE, Li Q, Gordon SN, Klatt NR, Duan L, et al (2009) Global genomic analysis reveals rapid control of a robust innate response in SIV-infected sooty mangabeys. J Clin Invest 119: 3556–3572. 19959874 10.1172/JCI40115 PMC2786806

[35] Campillo-Gimenez L, Laforge M, Fay M, Brussel A, Cumont MC, et al (2010) Nonpathogenesis of simian immunodeficiency virus infection is associated with reduced inflammation and recruitment of plasmacytoid dendritic cells to lymph nodes, not to lack of an interferon type I response, during the acute phase. J Virol 84: 1838–1846. 19939930 10.1128/JVI.01496-09 PMC2812402

[36] Jacquelin B, Mayau V, Targat B, Liovat AS, Kunkel D, et al (2009) Nonpathogenic SIV infection of African green monkeys induces a strong but rapidly controlled type I IFN response. J Clin Invest 119: 3544–3555. 19959873 10.1172/JCI40093 PMC2786805

[37] Harris LD, Tabb B, Sodora DL, Paiardini M, Klatt NR, et al (2010) Downregulation of Robust Acute Type I Interferon Responses Distinguishes Nonpathogenic Simian Immunodeficiency Virus (SIV) Infection of Natural Hosts from Pathogenic SIV Infection of Rhesus Macaques. J Virol 84: 7886–7891. 20484518 10.1128/JVI.02612-09 PMC2897601

[38] Diop OM, Ploquin MJ, Mortara L, Faye A, Jacquelin B, et al (2008) Plasmacytoid dendritic cell dynamics and alpha interferon production during Simian immunodeficiency virus infection with a nonpathogenic outcome. J Virol 82: 5145–5152. 18385227 10.1128/JVI.02433-07 PMC2395206

[39] Pereira LE, Johnson RP, Ansari AA (2008) Sooty mangabeys and rhesus macaques exhibit significant divergent natural killer cell responses during both acute and chronic phases of SIV infection. Cellular Immunology 254: 10–19. 18640666 10.1016/j.cellimm.2008.06.006

[40] Lederer S, Favre D, Walters KA, Proll S, Kanwar B, et al (2009) Transcriptional profiling in pathogenic and non-pathogenic SIV infections reveals significant distinctions in kinetics and tissue compartmentalization. PLoS Pathog 5: e1000296. 19214219 10.1371/journal.ppat.1000296 PMC2633618

[41] Meythaler M, Martinot A, Wang Z, Pryputniewicz S, Kasheta M, et al (2009) Differential CD4+ T-Lymphocyte Apoptosis and Bystander T-Cell Activation in Rhesus Macaques and Sooty Mangabeys during Acute Simian Immunodeficiency Virus Infection. J Virol 83: 572–583. 18987149 10.1128/JVI.01715-08 PMC2612394

[42] Favre D, Lederer S, Kanwar B, Ma ZM, Proll S, et al (2009) Critical loss of the balance between Th17 and T regulatory cell populations in pathogenic SIV infection. PLoS Pathog 5: e1000295. 19214220 10.1371/journal.ppat.1000295 PMC2635016

[43] Stacey AR, Norris PJ, Qin L, Haygreen EA, Taylor E, et al (2009) Induction of a striking systemic cytokine cascade prior to peak viraemia in acute human immunodeficiency virus type 1 infection, in contrast to more modest and delayed responses in acute hepatitis B and C virus infections. J Virol 3719–3733. 19176632 10.1128/JVI.01844-08 PMC2663284

[44] Fukazawa Y, Park H, Cameron MJ, Lefebvre F, Lum R, et al (2012) Lymph node T cell responses predict the efficacy of live attenuated SIV vaccines. Nat Med 18: 1673–1681. 22961108 10.1038/nm.2934 PMC3493820

[45] Buettner M, Bode U (2012) Lymph node dissection – understanding the immunological function of lymph nodes. Clinical & Experimental Immunology 169: 205–212. 22861359 10.1111/j.1365-2249.2012.04602.x PMC3444996

[46] Pandrea I, Kornfeld C, Ploquin MJ, Apetrei C, Faye A, et al (2005) Impact of viral factors on very early in vivo replication profiles in simian immunodeficiency virus SIVagm-infected African green monkeys. J Virol 79: 6249–6259. 15858009 10.1128/JVI.79.10.6249-6259.2005 PMC1091729

[47] Wijewardana V, Soloff AC, Liu X, Brown KN, Barratt-Boyes SM (2010) Early Myeloid Dendritic Cell Dysregulation is Predictive of Disease Progression in Simian Immunodeficiency Virus Infection. PLoS Pathog 6: e1001235. 21203477 10.1371/journal.ppat.1001235 PMC3009592

[48] Wijewardana V, Kristoff J, Xu C, Ma D, Haret-Richter G, et al (2013) Kinetics of myeloid dendritic cell trafficking and activation: impact on progressive, nonprogressive and controlled SIV infections. PLoS Pathog 9: e1003600. 24098110 10.1371/journal.ppat.1003600 PMC3789723

[49] Brown KN, Trichel A, Barratt-Boyes SM (2007) Parallel Loss of Myeloid and Plasmacytoid Dendritic Cells from Blood and Lymphoid Tissue in Simian AIDS. The Journal of Immunology 178: 6958–6967. 17513745 10.4049/jimmunol.178.11.6958

[50] Dillon SM, Robertson KB, Pan SC, Mawhinney S, Meditz AL, et al (2008) Plasmacytoid and myeloid dendritic cells with a partial activation phenotype accumulate in lymphoid tissue during asymptomatic chronic HIV-1 infection. J Acquir Immune Defic Syndr 48: 1–12. 18300699 10.1097/QAI.0b013e3181664b60 PMC2529020

[51] Dambaeva SV, Breburda EE, Durning M, Garthwaite MA, Golos TG (2009) Characterization of decidual leukocyte populations in cynomolgus and vervet monkeys. J Reprod Immunol 80: 57–69. 19398130 10.1016/j.jri.2008.12.006 PMC3076217

[52] Webster RL, Johnson RP (2005) Delineation of multiple subpopulations of natural killer cells in rhesus macaques. Immunology 115: 206–214. 15885126 10.1111/j.1365-2567.2005.02147.x PMC1782152

[53] Cooper MA, Fehniger TA, Caligiuri MA (2001) The biology of human natural killer-cell subsets. Trends Immunol 22: 633–640. 11698225 10.1016/s1471-4906(01)02060-9

[54] Cousens LP, Peterson R, Hsu S, Dorner A, Altman JD, et al (1999) Two roads diverged: interferon alpha/beta- and interleukin 12-mediated pathways in promoting T cell interferon gamma responses during viral infection. J Exp Med 189: 1315–1328. 10209048 10.1084/jem.189.8.1315 PMC2193028

[55] Giavedoni LD, Velasquillo MC, Parodi LM, Hubbard GB, Hodara VL (2000) Cytokine expression, natural killer cell activation, and phenotypic changes in lymphoid cells from rhesus macaques during acute infection with pathogenic simian immunodeficiency virus. J Virol 74: 1648–1657. 10644334 10.1128/jvi.74.4.1648-1657.2000 PMC111639

[56] Milush JM, Stefano-Cole K, Schmidt K, Durudas A, Pandrea I, et al (2007) Mucosal innate immune response associated with a timely humoral immune response and slower disease progression after oral transmission of simian immunodeficiency virus to rhesus macaques. J Virol 81: 6175–6186. 17428863 10.1128/JVI.00042-07 PMC1900075

[57] Vanderford TH, Slichter C, Rogers KA, Lawson BO, Obaede R, et al (2012) Treatment of SIV-infected sooty mangabeys with a type-I IFN agonist results in decreased virus replication without inducing hyperimmune activation. Blood 119: 5750–5757. 22550346 10.1182/blood-2012-02-411496 PMC3382934

[58] Wills RJ, Spiegel HE, Soike KF (1984) Pharmacokinetics of recombinant alpha A interferon following I.V. infusion and bolus, I.M., and P.O. administrations to African green monkeys. J Interferon Res 4: 399–409. 6491397 10.1089/jir.1984.4.399

[59] Jazayeri JA, Carroll GJ (2008) Fc-based cytokines : prospects for engineering superior therapeutics. BioDrugs 22: 11–26. 18215087 10.2165/00063030-200822010-00002

[60] Omer FM, de Souza JB, Riley EM (2003) Differential induction of TGF-beta regulates proinflammatory cytokine production and determines the outcome of lethal and nonlethal Plasmodium yoelii infections. J Immunol 171: 5430–5436. 14607947 10.4049/jimmunol.171.10.5430

[61] Zuniga EI, Liou LY, Mack L, Mendoza M, Oldstone MB (2008) Persistent virus infection inhibits type I interferon production by plasmacytoid dendritic cells to facilitate opportunistic infections. Cell Host Microbe 4: 374–386. 18854241 10.1016/j.chom.2008.08.016 PMC2875928

[62] Asmuth DM, Abel K, George MD, Dandekar S, Pollard RB, et al (2008) Pegylated interferon-alpha 2a treatment of chronic SIV-infected macaques. J Med Primatol 37: 26–30. 18199069 10.1111/j.1600-0684.2007.00221.x PMC2674790

[63] Goujard C, Emilie D, Roussillon C, Godot V, Rouzioux C, et al (2012) Continuous versus intermittent treatment strategies during primary HIV-1 infection: the randomized ANRS INTERPRIM Trial. Aids 26: 1895–1905. 22842994 10.1097/QAD.0b013e32835844d9

[64] Azzoni L, Foulkes AS, Papasavvas E, Mexas AM, Lynn KM, et al (2013) Reply to zur Wiesch and van Lunzen. Journal of Infectious Diseases 208: 363. 23570845 10.1093/infdis/jit160

[65] Durudas A, Milush JM, Chen HL, Engram JC, Silvestri G, et al (2009) Elevated levels of innate immune modulators in lymph nodes and blood are associated with more-rapid disease progression in simian immunodeficiency virus-infected monkeys. J Virol 83: 12229–12240. 19759147 10.1128/JVI.01311-09 PMC2786739

[66] Simmons RP, Scully EP, Groden EE, Arnold KB, Chang JJ, et al (2013) HIV-1 infection induces strong production of IP-10 through TLR7/9-dependent pathways. Aids 27: 2505–2517. 24096630 10.1097/01.aids.0000432455.06476.bc PMC4288813

[67] Kamat A, Misra V, Cassol E, Ancuta P, Yan Z, et al (2012) A plasma biomarker signature of immune activation in HIV patients on antiretroviral therapy. PLoS ONE 7: e30881. 22363505 10.1371/journal.pone.0030881 PMC3281899

[68] Keating SM, Golub ET, Nowicki M, Young M, Anastos K, et al (2011) The effect of HIV infection and HAART on inflammatory biomarkers in a population-based cohort of women. Aids 25: 1823–1832. 21572306 10.1097/QAD.0b013e3283489d1f PMC3314300

[69] Hilkens CM, Schlaak JF, Kerr IM (2003) Differential responses to IFN-alpha subtypes in human T cells and dendritic cells. J Immunol 171: 5255–5263. 14607926 10.4049/jimmunol.171.10.5255

[70] Kanda N, Shimizu T, Tada Y, Watanabe S (2007) IL-18 enhances IFN-gamma-induced production of CXCL9, CXCL10, and CXCL11 in human keratinocytes. Eur J Immunol 37: 338–350. 17274000 10.1002/eji.200636420

[71] Yeruva S, Ramadori G, Raddatz D (2008) NF-kappaB-dependent synergistic regulation of CXCL10 gene expression by IL-1beta and IFN-gamma in human intestinal epithelial cell lines. Int J Colorectal Dis 23: 305–317. 18046562 10.1007/s00384-007-0396-6 PMC2225996

[72] Pulit-Penaloza JA, Scherbik SV, Brinton MA (2012) Type 1 IFN-independent activation of a subset of interferon stimulated genes in West Nile virus Eg101-infected mouse cells. Virology 425: 82–94. 22305622 10.1016/j.virol.2012.01.006 PMC3288888

[73] Rustagi A, Gale M Jr (2014) Innate Antiviral Immune Signaling, Viral Evasion and Modulation by HIV-1. Journal of Molecular Biology 426: 1161–1177. 24326250 10.1016/j.jmb.2013.12.003 PMC7425209

[74] Hasan M, Koch J, Rakheja D, Pattnaik AK, Brugarolas J, et al (2013) Trex1 regulates lysosomal biogenesis and interferon-independent activation of antiviral genes. Nat Immunol 14: 61–71. 23160154 10.1038/ni.2475 PMC3522772

[75] Malcolm KC, Worthen GS (2003) Lipopolysaccharide Stimulates p38-dependent Induction of Antiviral Genes in Neutrophils Independently of Paracrine Factors. Journal of Biological Chemistry 278: 15693–15701. 12595530 10.1074/jbc.M212033200

[76] Brenchley JM, Price DA, Schacker TW, Asher TE, Silvestri G, et al (2006) Microbial translocation is a cause of systemic immune activation in chronic HIV infection. Nat Med 12: 1365–1371. 17115046 10.1038/nm1511

[77] Handley SA, Thackray LB, Zhao G, Presti R, Miller AD, et al (2012) Pathogenic simian immunodeficiency virus infection is associated with expansion of the enteric virome. Cell 151: 253–266. 23063120 10.1016/j.cell.2012.09.024 PMC3490196

[78] Aguet M, Grobke M, Dreiding P (1984) Various human interferon alpha subclasses cross-react with common receptors: their binding affinities correlate with their specific biological activities. Virology 132: 211–216. 6320534 10.1016/0042-6822(84)90105-3

[79] Gibbert K, Joedicke JJ, Meryk A, Trilling M, Francois S, et al (2012) Interferon-alpha Subtype 11 Activates NK Cells and Enables Control of Retroviral Infection. PLoS Pathogens 8: e1002868. 22912583 10.1371/journal.ppat.1002868 PMC3415439

[80] Moll HP, Maier T, Zommer A, Lavoie T, Brostjan C (2011) The differential activity of interferon-alpha subtypes is consistent among distinct target genes and cell types. Cytokine 53: 52–59. 20943413 10.1016/j.cyto.2010.09.006 PMC3020287

[81] Silvestri G, Fedanov A, Germon S, Kozyr N, Kaiser WJ, et al (2005) Divergent Host Responses during Primary Simian Immunodeficiency Virus SIVsm Infection of Natural Sooty Mangabey and Nonnatural Rhesus Macaque Hosts. J Virol 79: 4043–4054. 15767406 10.1128/JVI.79.7.4043-4054.2005 PMC1061583

[82] MacDonald KPA, Munster DJ, Clark GJ, Dzionek A, Schmitz J, et al (2002) Characterization of human blood dendritic cell subsets. Blood 100: 4512–4520. 12393628 10.1182/blood-2001-11-0097

[83] Piccioli D, Tavarini S, Borgogni E, Steri V, Nuti S, et al (2007) Functional specialization of human circulating CD16 and CD1c myeloid dendritic-cell subsets. Blood 109: 5371–5379. 17332250 10.1182/blood-2006-08-038422

[84] O'Brien M, Manches O, Sabado RL, Jimenez Baranda S, Wang Y, et al (2011) Spatiotemporal trafficking of HIV in human plasmacytoid dendritic cells defines a persistently IFN-alpha-producing and partially matured phenotype. J Clin Invest 121: 1088–1101. 21339641 10.1172/JCI44960 PMC3049388

[85] Bruel T, Dupuy S, Demoulins T, Rogez-Kreuz C, Dutrieux J, et al (2014) Plasmacytoid dendritic cell dynamics tune interferon-alfa production in SIV-infected cynomolgus macaques. PLoS Pathog 10: e1003915. 24497833 10.1371/journal.ppat.1003915 PMC3907389

[86] Nguyen KB, Salazar-Mather TP, Dalod MY, Van Deusen JB, Wei XQ, et al (2002) Coordinated and distinct roles for IFN-alpha beta, IL-12, and IL-15 regulation of NK cell responses to viral infection. J Immunol 169: 4279–4287. 12370359 10.4049/jimmunol.169.8.4279

[87] Martinot AJ, Meythaler M, Pozzi L-A, Dalecki Boisvert K, Knight H, et al (2013) Acute SIV Infection in Sooty Mangabey Monkeys Is Characterized by Rapid Virus Clearance from Lymph Nodes and Absence of Productive Infection in Germinal Centers. PLoS ONE 8: e57785. 23472105 10.1371/journal.pone.0057785 PMC3589484

[88] Brenchley JM, Vinton C, Tabb B, Hao XP, Connick E, et al (2012) Differential infection patterns of CD4+ T cells and lymphoid tissue viral burden distinguish progressive and nonprogressive lentiviral infections. Blood 120: 4172–4181. 22990012 10.1182/blood-2012-06-437608 PMC3501715

[89] Meythaler M, Wang Z, Martinot A, Pryputniewicz S, Kasheta M, et al (2011) Early induction of polyfunctional simian immunodeficiency virus (SIV)-specific T lymphocytes and rapid disappearance of SIV from lymph nodes of sooty mangabeys during primary infection. J Immunol 186: 5151–5161. 21441446 10.4049/jimmunol.1004110 PMC3130630

[90] Diop OM, Gueye A, Dias-Tavares M, Kornfeld C, Faye A, et al (2000) High levels of viral replication during primary simian immunodeficiency virus SIVagm infection are rapidly and strongly controlled in African green monkeys. J Virol 74: 7538–7547. 10906207 10.1128/jvi.74.16.7538-7547.2000 PMC112274

[91] Ploquin MJ, Jacquelin B, Jochems SP, Barre-Sinoussi F, Muller-Trutwin MC (2012) Innate immunity in the control of HIV/AIDS: recent advances and open questions. Aids 26: 1269–1279. 22472855 10.1097/QAD.0b013e328353e46b

