# Supplementary figures and images for: Innate Immune Responses and Rapid Control of Inflammation in African Green Monkeys Treated or Not with Interferon-Alpha during Primary SIVagm Infection
Source: PLoS Pathog. 2014 Jul 3;10(7):e1004241. doi: 10.1371/journal.ppat.1004241 (PMC4081777; doi:10.1371/journal.ppat.1004241)

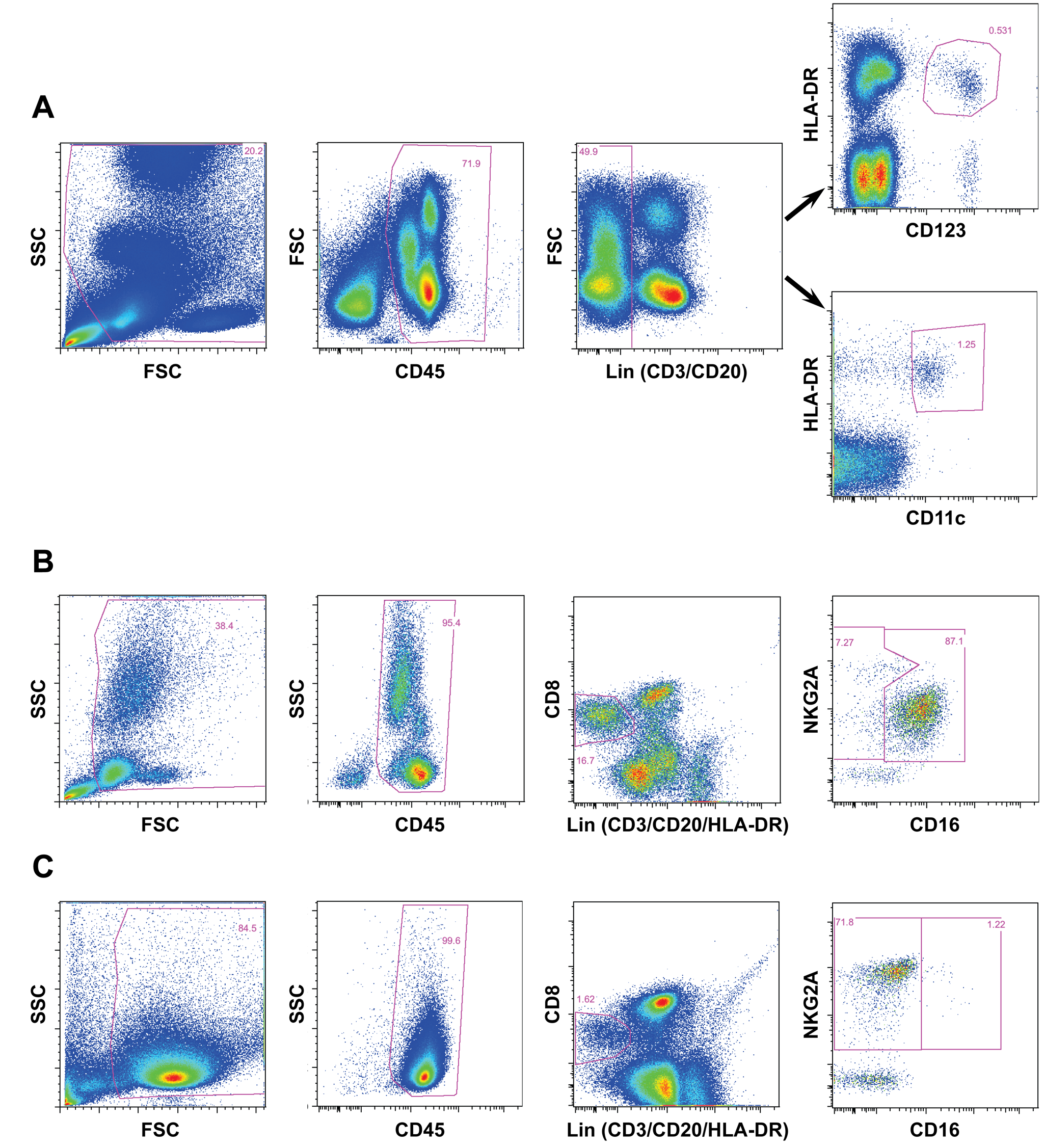

Supplement: Figure S1 — Flow-cytometric gating strategy. Flow-cytometric gating strategy used to identify (A) pDCs and mDCs, (B) CD16+/− NK cells in blood and (C) CD16− NK cells in LNs. Representative flow-cytometric analysis with whole blood (A, B) and LN cells (C) from a healthy AGM. (TIF) [file ppat.1004241.s001.tif]

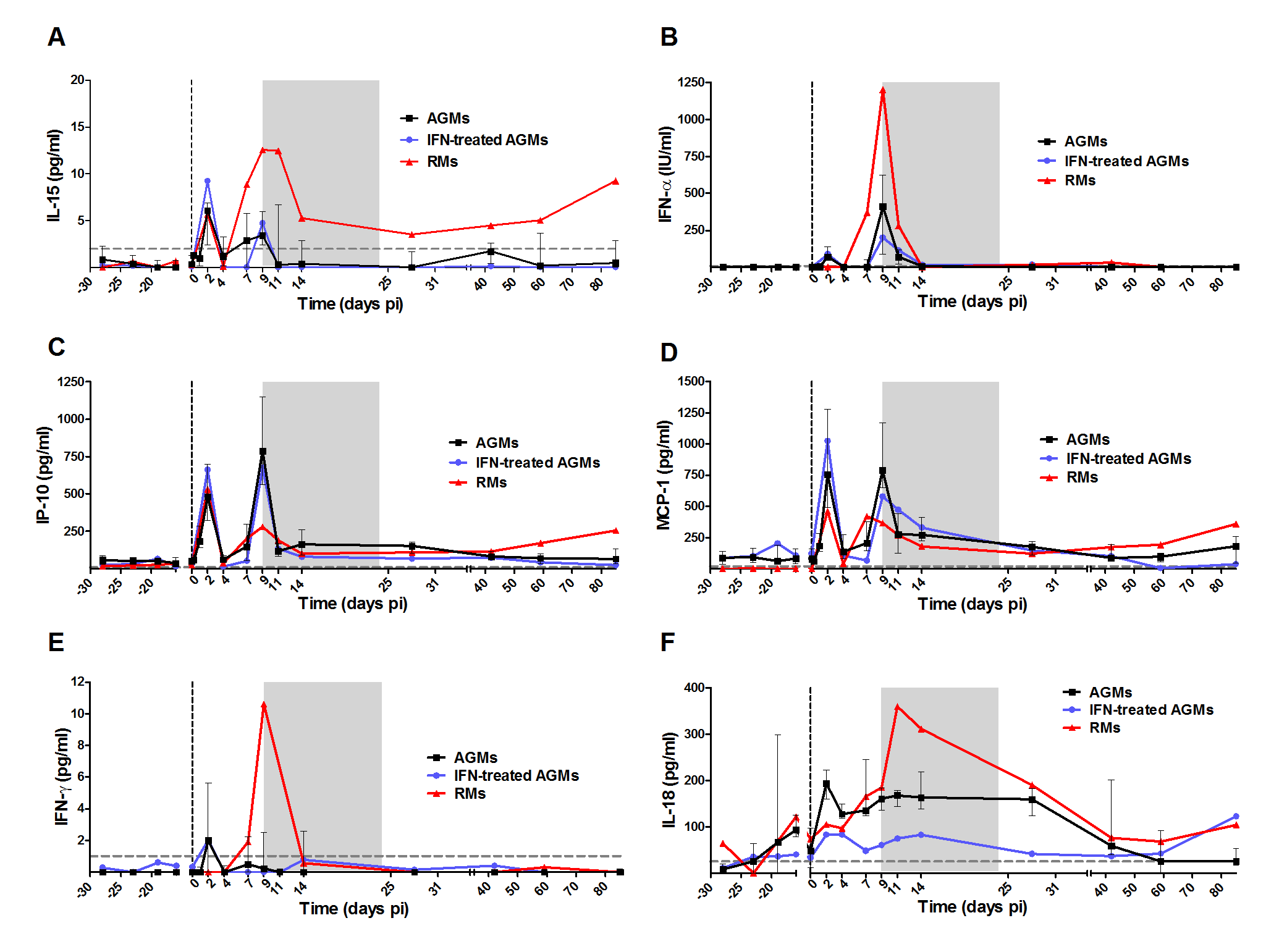

Supplement: Figure S3 — Comparison of the cytokine levels and kinetics in treated and untreated AGMs with those of rhesus macaques. Plasma levels of cytokines in 14 AGMs infected with SIVagm.sab92018 (black), the 2 IFN-α treated AGMs (blue) and 2 rhesus macaques infected with SIVmac251 (RM) (red). The levels of 4 early cytokines (A) IL-15, (B) IFN-α, (C) IP-10, (D) MCP-1 and 2 cytokines reported to appear later in SIVmac/HIV-1 primary infection (E) IFN-γ and (F) IL-18 (n = 6 AGMs) were determined. Data are presented as medians and interquartile ranges for untreated AGMs and as median only for the 2 treated AGMs and the 2 RMs. Day zero represents the median of all the time points before infection. All the AGMs and RMs were infected on day zero. The grey area indicates the period of IFN-α treatment. (TIF) [file ppat.1004241.s003.tif]

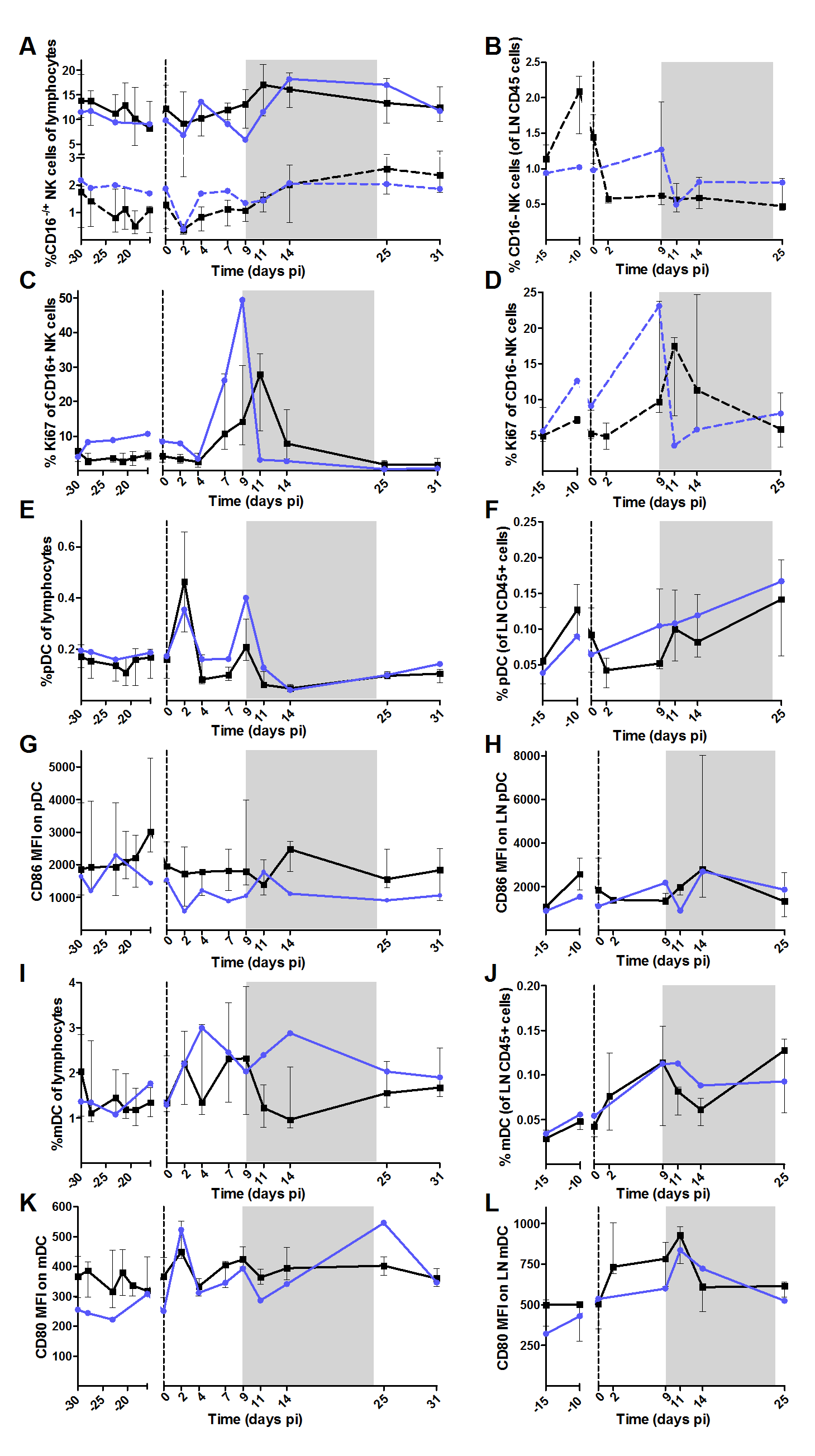

Supplement: Figure S4 — High dose of recombinant IFN-α injection during primary SIVagm infection does not affect innate immune cells. Analysis of the effect of high dose IFN-α injection in vivo on NK cells (A, B) frequencies and (C, D) activation (Ki-67%), on pDCs (E, F) frequencies and (G, H) maturation (CD86 mfi) and on mDCs (I, J) frequencies and (K, L) maturation (CD80 mfi), in blood (left panels) and LNs (right panels). Data are presented as medians and interquartile ranges for untreated animals (black) and median only for treated animals (blue). Day zero represents the median of all the time points before infection. The grey area indicates the period of treatment. The medians of treated animals were inside the interquartile range of the control untreated animals and were thus considered not different. (TIF) [file ppat.1004241.s004.tif]

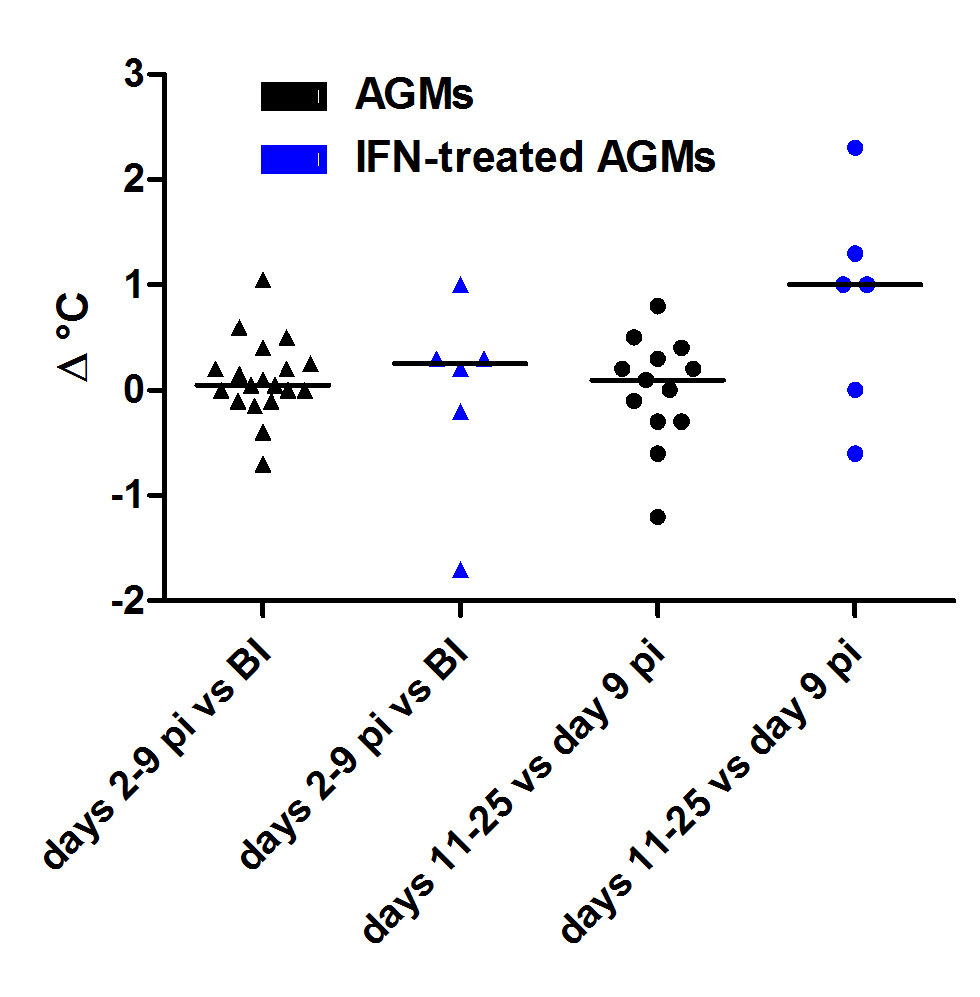

Supplement: Figure S5 — Effect of injection with high doses of recombinant IFN-α during primary SIVagm infection on body temperature. Body temperature changes as compared to temperature either before infection (BI) or before treatment (day 9 p.i.) for the 2 treated AGMs (blue) and 6 untreated AGMs (black). The temperature values after infection but before IFN-α-treatment correspond to time points between days 2 and 9 pi and are shown on the left. In the IFN-α-treated animals (between days 11 and 25 pi) on the right, the changes are indicated relative to the time point before treatment initiation (day 9 pi) and compared to the body temperature of the 6 untreated AGMs during the same time period. The median is indicated. (TIF) [file ppat.1004241.s005.tif]
